# Supplementary material for: EGFR-targeted fluorescence molecular imaging for intraoperative margin assessment in oral cancer patients: a phase II trial
Source: Nat Commun. 2023 Aug 16;14:4952. doi: 10.1038/s41467-023-40324-8 (PMC10432510; doi:10.1038/s41467-023-40324-8)
Supplement: Supplementary file 1 — Supplementary Information [file 41467_2023_40324_MOESM1_ESM.pdf]

Supplementary Results

Survival statistics

In 63 patients (two patients participated twice), we report a median follow up of 2.96 (0.61 to 4.07) years. Twenty-three patients reported with local recurrence (n=9), secondary primary tumors (n=9) or late lymph node metastasis (n=6) occurring within 0.91 (0.04 – 2.45) years. Considering the differences in follow-up time, overall disease-free survival (DFS) was 1.92 (0.04 to 4.03) years. Looking at margin status and survival, DFS was 2.57 (0.48 to 4.03) years for tumor free margins, 1.95 (0.14 to 3.80) for close margins and 0.85 (0.04 to 3.74) for tumor-positive margins. Eighteen patients have deceased, of which 14 due to oral squamous cell carcinoma. Of these 14 patients, eight had a tumor-positive margin, four a close margin and two a tumor-free margin during the initial surgery.

		Event	AE/SAE	Grade	Intervention
**Related**	1	Allergic reaction	AE	1	Rescue medication
	2	Flush	AE	1	None
	3	Chills	AE	1	None
	4	Skin rash	AE	1	None
	5	Skin rash	AE	1	I.V. Dexamethasone 4 mg
	6	Icterus	AE	1	Switch of antibiotics
	7	Vasovagal collapse	AE	1	Tracer abortion
	8	Bronchospasm	AE	2	Salbutamol nebulization
	9	Anaphylaxis	SAE	3	Rescue medication
	10	Anaphylaxis	SAE	3	Rescue medication
**Unrelated**	1	Hypotension during OR	AE	1	I.V. fluid suppletion
	2	Wound haemorrhage	AE	1	None
	3	Oliguria	AE	1	None
	4	Melena	AE	1	None
	5	Wound infection	AE	1	Rinsing with chlorhexidine
	6	Hyperkalaemia	AE	1	Restart hydrochlorothiazide
	7	Icterus	AE	1	Switch of antibiotics
	8	Haematoma neck	AE	3	Surgical relief
	9	Neck abscess	AE	3	Incision and drainage
	10	Neck abscess	AE	3	Incision and drainage
	11	Postoperative Hb drop	AE	3	Packed cell administration
	12	Syncope	SAE	3	None
	13	Acute coronary syndrome	SAE	3	ICU administration
	14	Haemorrhage neck	SAE	3	Reintervention OR
	15	Collapse, suspicion TIA	SAE	3	Hospital administration
	16	Failure of transplant	SAE	3	Reintervention
	17	Failure of transplant	SAE	3	Reintervention
	18	Failure of transplant	SAE	3	Reintervention
	19	Failure of transplant	SAE	3	Reintervention
	20	Cardiac arrest	SAE	4	Resuscitation

Supplementary Table 1: Overview of all adverse events. Abbreviations: AE, Adverse event; SAE, serious adverse event; I.V., Intravenous; ICU, Intensive care unit; OR, Operating room.

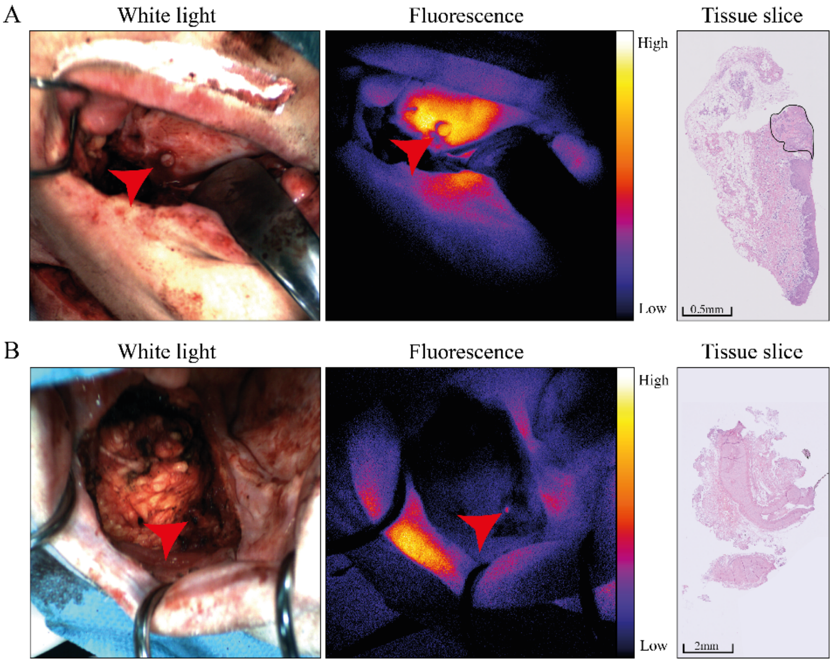

Supplementary Figure 1: Detection of satellite lesions using fluorescence molecular imaging. A) White light imaging shows apparently normal epithelium, but increased fluorescence is observed at this location. Punch biopsy (red arrowhead) reveals invasive carcinoma (solid black line). B) A fluorescent lesion is observed in the woundbed (red arrowhead), and palpation or white light imaging did not reveal the nature of the tissue. A biopsy was obtained, which showed artery tissue.

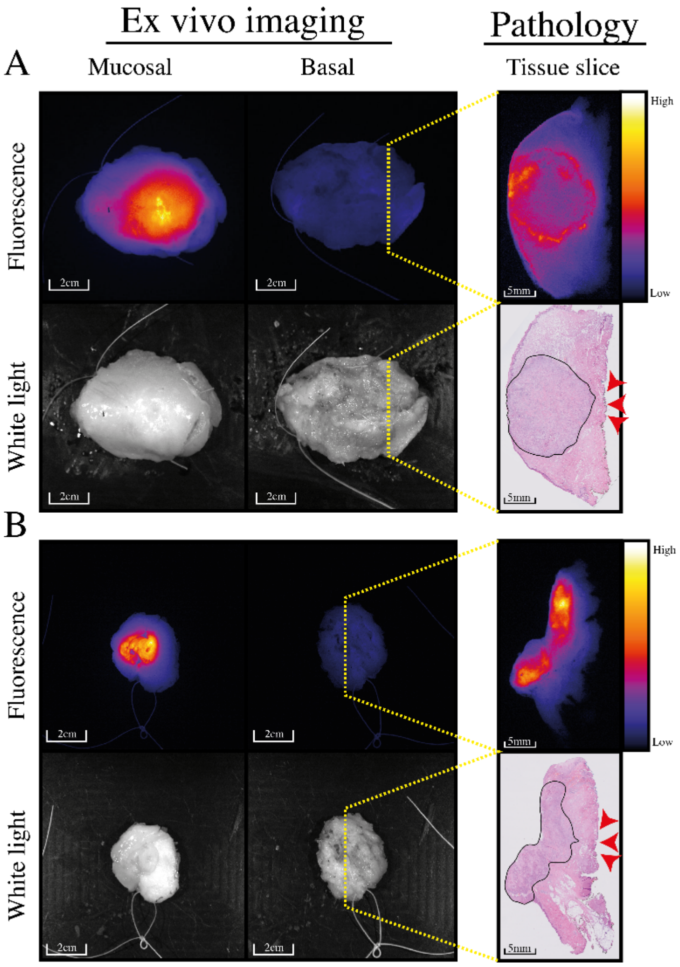

Supplementary Figure 2: Representative examples of false negative results. A) A false negative fluorescence result corresponding to a close margin of 1.9mm (red arrowheads), in a tumor (solid black line) with a large necrotic core with limited viable tumor cells on haematoxylin & eosin histochemistry. No fluorescence is observed in the core of the tumor. B) A false negative margin of 2.2mm (red arrowheads), for which immunohistochemistry cannot provide an explanation for the absence of fluorescence signal in the margin, again the tumor is demarcated with a solid black line on tissue slides.

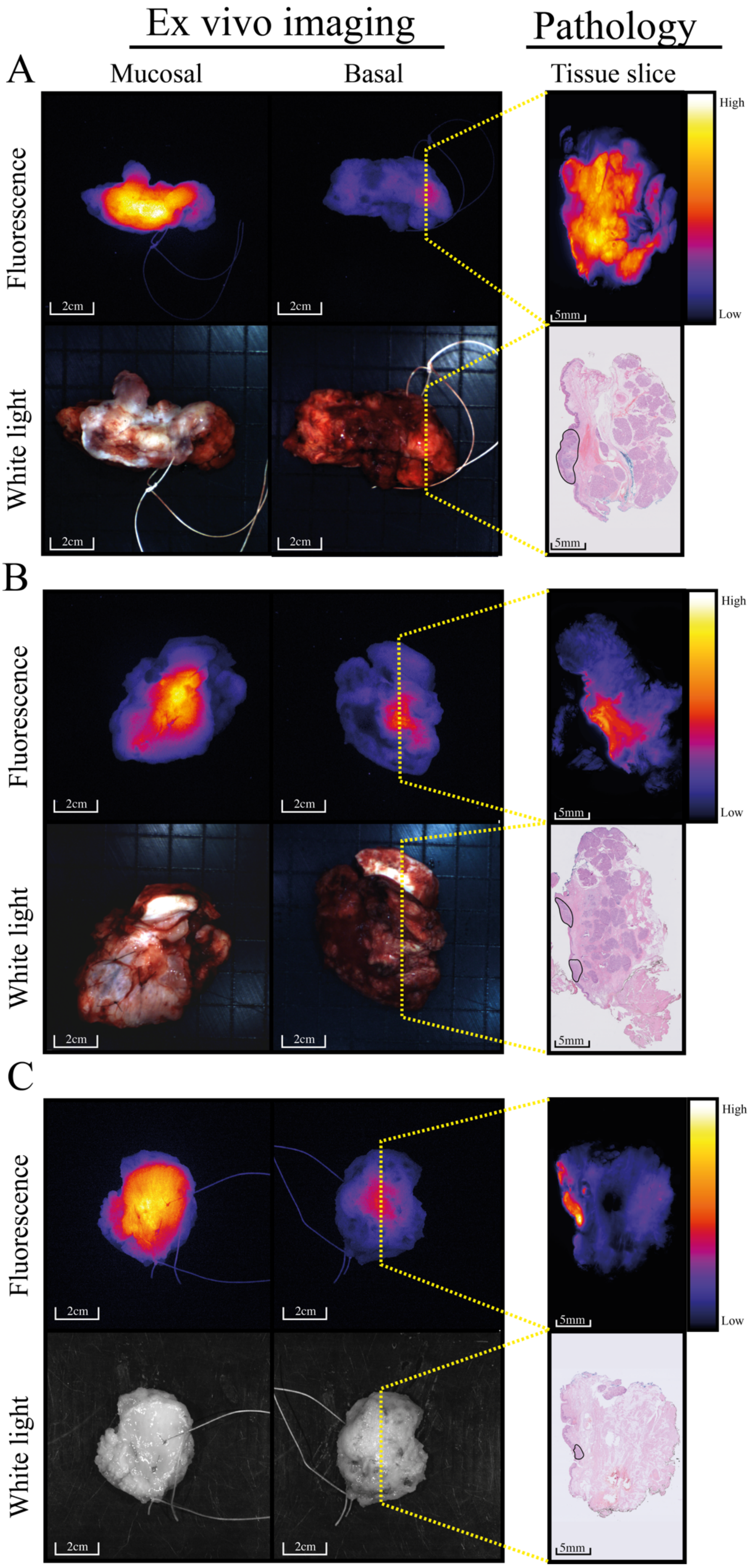

Ex vivo imaging
Pathology
A
Mucosal
Basal
Tissue slice
Fluorescence
White light
High
Low
2cm
5mm
B
C

Supplementary Figure 3: Representative examples of false positive results. A) A false positive result due to salivary gland tissue in the margin, where we also observe fluorescence signal in the salivary gland tissue. In B) the salivary gland does not correspond with fluorescence signal on cross-sectional imaging. C) A false positive fluorescent lesion in the margin of a very small tumor, which cannot be explained by histopathology. Tumors are demarcated with a solid black line on tissue slides.

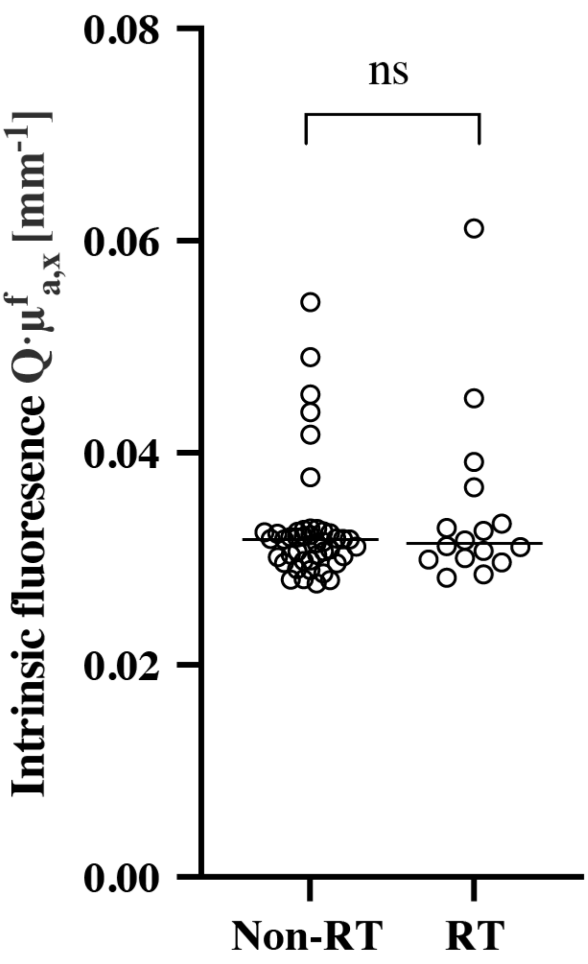

Supplementary Figure 4: Influence of previous irradiation on intrinsic fluorescence signal. No difference in intrinsic fluorescence signal is observed between patients who had received radiotherapy previously (n = 16, 3.1 (2.8-6.1)x10^{-2} mm^{-1}) in the head and neck area

and patients who had not (n = 47, 3.2 (2.8–5.4)x10^{-2} mm^{-1}, two sided p = 0.85) using Mann-Whitney test. Source data are provided as a Source Data file. Abbreviations: RT, Radiotherapy.

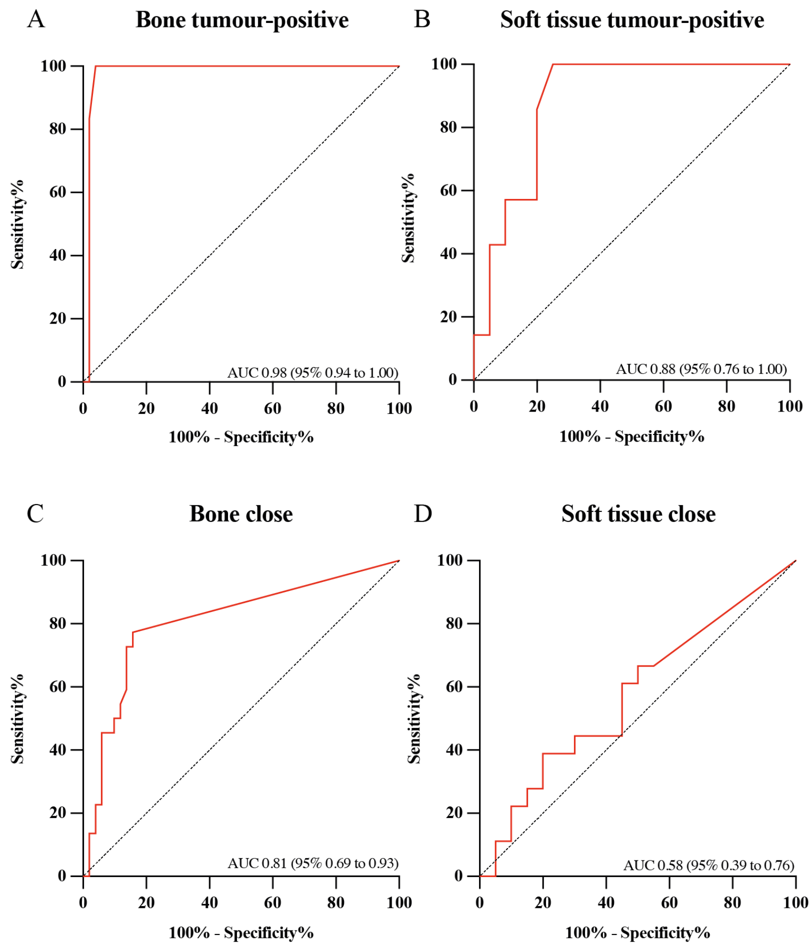

Supplementary Figure 5: Receiver operating characteristics curves of specimens with and without bone. For tumor-positive margins (Panels A & B) the AUCs of specimen containing bone (n = 29) are compared to specimen not containing bone (n = 37) using Z-scores, which are not significantly different (z = 1.50, two sided p = 0.067). The AUC for the detection of close margins is significantly larger in the specimen including bone (Panels C + D) (z = 3.28, two sided p = 0.005). Source data are provided as a Source Data file. Abbreviations: AUC, Area under the curve; CI, Confidence interval.

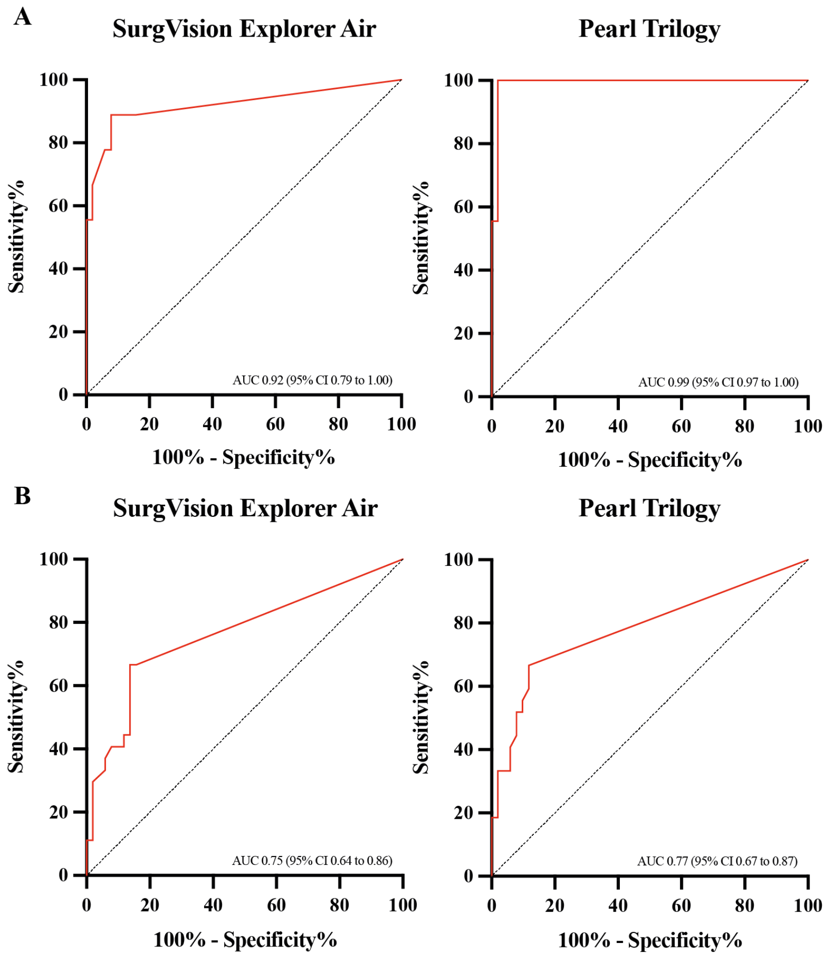

Supplementary Figure 6: Receiver operating characteristics curves of the Pearl-Trilogy® and the SurgVision explorer Air®. ROC curves from planes images by both the SurgVision Explorer Air® and the Pearl-Trilogy® of tumor-positive margins (N = 9) (Panel A) and close margins (N = 27) (Panel B) show no statistical different AUCs (bootstrap p=0.11 and p=0.48, respectively). Source data are provided as a Source Data file. Abbreviations: ROC, Receiver operating characteristics; AUC, Area under the curve; CI, Confidence interval.

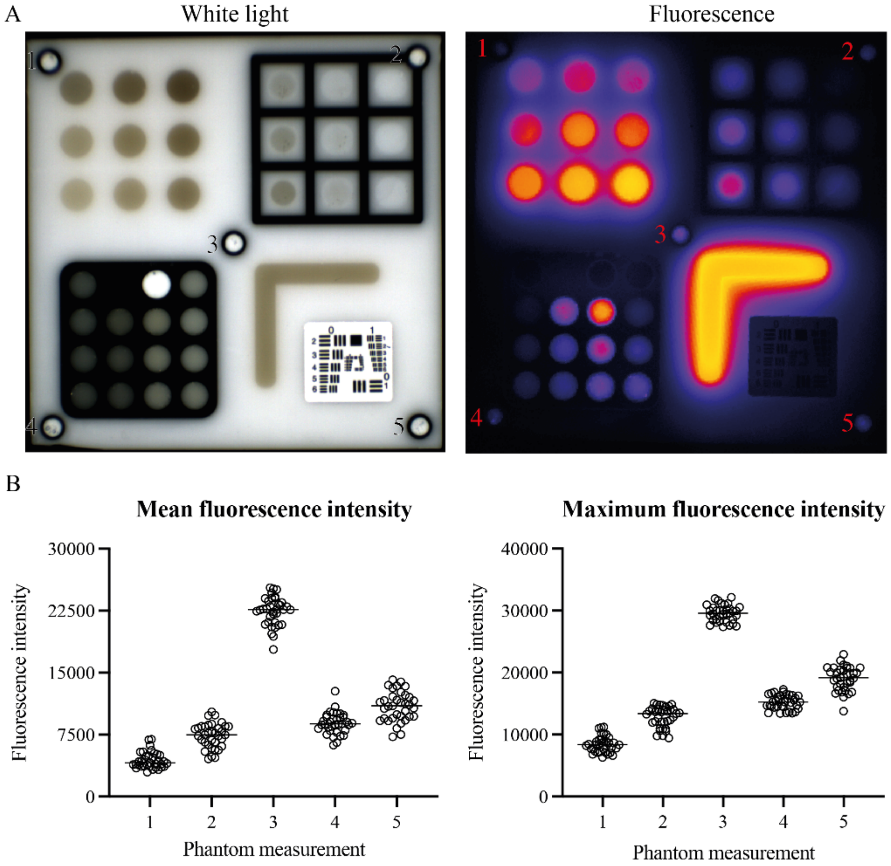

Supplementary Figure 7: Pre-operative phantom measurements. A.) White light and fluorescence image of the phantom for benchmarking fluorescence cameras[1]. B.) Segmentation of various locations on the fluorescence phantom (180 measurements from 36 images) illustrates an average standard deviation of 10%, partly explained by differences in manual segmentation of the region of interest. Source data are provided as a Source Data file.

Supplementary References

1. Gorpas, D. *et al.* Multi-Parametric Standardization of Fluorescence Imaging Systems Based on a Composite Phantom. *Ieee T Bio-med Eng* 67, 185–192 (2019).

Supplementary note

IMAGE GUIDED SURGERY FOR MARGIN ASSESSMENT OF HEAD AND NECK CANCER USING CETUXIMAB-IRDYE800CW CONJUGATE (ICON)

A single center, prospective, cross sectional, diagnostic study.

Clinical study protocol

Authors: University Medical Center Groningen (UMCG)

Version: 4.0

Date: April 2017

Short Title: EGFR-Targeted Near-Infrared Fluorescence Surgery in HNSCC patient

Protocol IDs:

CCMO: 58585.042.16

ABR: 58585

EurdraCT: 2016-002726-37

Study Center

University Medical Center Groningen (UMGG)

Hanzeplein 1

9713 GZ Groningen

The Netherlands

Contact Information

Dr. M.J.H. Witjes, MD, DDS, PhD

Dep of Oral and Maxillofacial Surgery, UMCG

Email: m.j.h.witjes@umcg.nl

+31-50-3613841 (Tel)

+31-50-3612831 (Fax)

PROTOCOL TITLE: Image guided surgery for margin assessment of head and neck Cancer using cetuximab-IRDye800CW cONjugate (ICON)

**Protocol ID**	**ICON**
**Short title**	**I**mage guided surgery for margin assessment of head and neck **C**ancer using cetuximab-IRDye800CW c**ON**jugate (ICON).
**EudraCT number**	2016-002726-37
**Version**	4.0
**Date**	**24-04-2017**
**Coordinating investigator/project leader**	Dr. M.J.H. Witjes<br>UMC Groningen, dept. of Oral and Maxilofacial Surgery<br>PO BOX 30.001 9700RB, Groningen<br>+31-50-3613841 or +31-50-3612831<br>**m.j.h.witjes@umcg.nl**
**Principal investigator(s) (in Dutch: hoofdonderzoeker/ uitvoerder)**	Dr. M.J.H. Witjes<br>UMC Groningen, dept. of Oral and Maxilofacial Surgery<br>PO BOX 30.001 9700RB, Groningen<br>+31-50-3613841 or +31-50-3612831<br>**m.j.h.witjes@umcg.nl**<br><br>Prof. dr. G.M. van Dam<br>UMC Groningen, dept. of Surgery, dept. of Nuclear Medicine and Molecular Imaging, Intensive Care<br>PO BOX 30.001, 9700RB, Groningen<br>+31-50-3610183<br>**g.m.van.dam@umcg.nl**
**Sponsor (in Dutch: verrichter/opdrachtgever)**	University Medical Center Gorningen<br>Hanzeplein 1<br>9713 GZ Groningen
**Subsidising party**	Dutch Cancer Society (in Dutch: KWF Nederland)

	**Grant Number KWF 2015-8084**
**Independent expert (s)**	Prof dr. GM Raghoebar, MD, DDS, PhD<br>UMC Groningen, dept. of Oral and Maxilofacial Surgery<br>PO BOX 30.001 9700RB, Groningen<br>The Netherlands
**Laboratory sites**	***Involved pathologists:*** Dr. B van der Vegt MD, PhD, Dr J.J. Doff, MD
**Pharmacy <*if applicable*>**	A. Jorritsma. PharmD, PhD, QP University Medical Center Groningen

PROTOCOL SIGNATURE SHEET

**Name**	**Signature**	**Date**
**Head of Department:**<br>Prof Dr FKL Spijkervet<br>Dep of Oral and Maxillofacial Surgery<br>PO Box 30.001<br>9700RB Groningen<br>The Netherlands		**24-4-17**
**leader/Principal Investigator:**<br>***Dr. M.J.H. Witjes***		**24-4-17**

TABLE OF CONTENTS

1. INTRODUCTION AND RATIONALE 14
1.1 The importance of a free surgical margin in head and neck squamous cell carcinoma 14
1.2 The rationale of intraoperative Near Infrared Fluorescent imaging in surgical removal of HNSCC 14
1.3 Intraoperative near infrared fluorescence imaging in cancer 15
1.4 The added value of spectroscopy in clinical decision making during fluorescence image-guided surgery 16
1.5 In vivo NIRF imaging: Clinical trials performed at the UMCG 17
1.6 Rationale for the use of cetuximab-IRDye800CW as fluorescent tracer in HNSCC 18
1.7 Dose selection for the cetuximab-IRDye800CW conjugate 21
2. OBJECTIVES 22
2.1 Primary objective Part 1 (dose finding) 22
2.2 Primary objective Part 2 (main study) 22
2.3 Secondary Objectives 23
2.4 End points Part 1 (dose finding) 23
2.5 End points Part 2 (main study) 23
3. STUDY DESIGN 24
3.1 Study design Part 1 (dose finding) 24
3.2 Study design part 2 25
4. STUDY POPULATION 26
4.1 Population (base) 26
4.2 Inclusion criteria 26
4.3 Exclusion criteria 26
4.4 Sample size calculation 27
4.4.1 Sample size calculation Part 1 (dose finding) 27
4.4.2 Sample size calculation Part 2 (main study) 27
5. TREATMENT OF SUBJECTS 28
5.1 Investigational product/treatment 28
6. INVESTIGATIONAL PRODUCT 28
6.1 Name and description of investigational product(s) 28
6.2 Summary of findings from non-clinical studies 29
6.3 Summary of findings from clinical studies 29
6.4 Summary of known and potential risks and benefits 29
6.5 Description and justification of route of administration and dosage 30
6.6 Dosages, dosage modifications and method of administration 30
6.7 Preparation and labeling of Investigational Medicinal Product 30
6.8 Drug accountability 31
7. Investigational medical device: Imaging devices 31
7.1 Name and description of non-investigational product(s) 31
7.1.1 SurgVision F2 multispectral imaging system 31

7.1.2 Flexible Nasopharyngeal endoscope ... 32
7.1.3 MDSFR/SFF spectroscope ... 32
7.2 Summary of findings from non-clinical studies ... 33
7.3 Summary of findings from clinical studies ... 33
7.4 Summary of known and potential risks and benefits ... 33
7.5 Description and justification of route of administration and dosage ... 34
7.6 Dosages, dosage modifications and method of administration ... 34
7.7 Preparation and labelling of Non Investigational Medicinal Product ... 34
7.8 Drug accountability ... 34
8. METHODS ... 35
8.1 Study parameters/endpoints ... 35
8.1.1 Main study parameter/endpoint ... 35
8.1.2 Other study parameters (if applicable) ... 35
8.2 Randomization, blinding and treatment allocation ... 35
8.3 Study procedures ... 36
8.4 Withdrawal of individual subjects ... 40
8.4.1 Specific criteria for withdrawal (if applicable) ... 40
8.5 Replacement of individual subjects after withdrawal ... 40
8.6 Follow-up of subjects withdrawn from treatment ... 40
8.7 Premature termination of the study ... 40
8.7.1 Termination based on safety aspects ... 40
8.7.2 Termination based on Cetuximab-IRDye800CW accumulation ... 40
8.7.3 Termination based on other aspects ... 41
9. SAFETY REPORTING ... 41
9.1 Temporary halt for reasons of subject safety ... 41
9.2 AEs, SAEs and SUSARs ... 41
9.2.1 Adverse events (AEs) ... 41
9.2.2 Serious adverse events (SAEs) ... 41
9.2.3 Suspected unexpected serious adverse reactions (SUSARs) ... 42
9.3 Annual safety report ... 43
9.4 Follow-up of adverse events ... 43
9.5 Data Safety Monitoring Board (DSMB) ... 43
10. STATISTICAL ANALYSIS ... 45
10.1 Descriptive statistics ... 45
10.2 Study parameters ... 45
10.3 Interim analysis ... 46
11. ETHICAL CONSIDERATIONS ... 46
11.1 Regulation statement ... 46
11.2 Recruitment and consent ... 46
11.3 Objection by minors or incapacitated subjects (if applicable) ... 47
11.4 Benefits and risks assessment, group relatedness ... 47
11.5 Compensation for injury ... 48
11.6 Incentives (if applicable) ... 48

12. ADMINISTRATIVE ASPECTS, MONITORING AND PUBLICATION 48
12.1 Handling and storage of data and documents 49
12.2 Monitoring and Quality Assurance 49
12.3 Amendments 50
12.4 Annual progress report 50
12.5 Temporary halt and (prematurely) end of study report 50
12.6 Public disclosure and publication policy 51
13. STRUCTURED RISK ANALYSIS 51
13.1 Potential issues of concern 51
13.2 Synthesis 54
14. REFERENCES 55

LIST OF ABBREVIATIONS AND RELEVANT DEFINITIONS

**ABR**	**ABR form, General Assessment and Registration form, is the application form that is required for submission to the accredited Ethics Committee (In Dutch, ABR = Algemene Beoordeling en Registratie)**
**AE**	**Adverse Event**
**AR**	**Adverse Reaction**
**CA**	**Competent Authority**
**CCMO**	**Central Committee on Research Involving Human Subjects; in Dutch: Centrale Commissie Mensgebonden Onderzoek**
**CRU**	**Clinical Research Unit**
**CV**	**Curriculum Vitae**
**DSMB**	**Data Safety Monitoring Board**
**EGFR**	**Epidermal Growth Factor Receptor**
**EPD**	**Electronic patients file (in Dutch: Electronisch Patienten Dossier)**
**EPR-effect**	**Enhanced Permeability and Retention effect**
**EU**	**European Union**
**EudraCT**	**European drug regulatory affairs Clinical Trials**
**FAP**	**Familial Adenomatous Polyposis**
**FOV**	**Field of view**
**FITC**	**Fluorescein Isothiocyanate**
**FR-a**	**Folate receptor-α**
**GCP**	**Good Clinical Practice**
**GMP**	**Good Manufacturing Practice**
**HNSCC**	**Head and Neck Squamous Cell Carcinoma**
**IB**	**Investigator’s Brochure**
**IC**	**Informed Consent**
**ICG**	**Indocyanine Green**
**IHC**	**Immunohistochemical**
**IMP**	**Investigational Medicinal Product**
**IMPD**	**Investigational Medicinal Product Dossier**
**MDSFR**	**Multidiameter single-fiber reflectance**
**METC**	**Medical research ethics committee (MREC); in Dutch: medisch ethische toetsing commissie (METC)**

**MFI**	**Mean Fluorescence Intensity**
**MAb**	**Monoclonal Antibodies**
**NIR**	**Near Infrared**
**OSCC**	**Oral Squamous Cell Carcinoma**
**PET**	**Positron emission tomography**
**PROFIT**	**PROfessionalization and Facilitation of Investigator initiated Trials**
**ROI**	**Region of Interest**
**(S)AE**	**(Serious) Adverse Event**
**SE-HPLC**	**Size-exclusion high-performance liquid chromatography**
**SFF**	**Single Fiber Fluorescence**
**SFR**	**Single Fiber Reflectance**
**SPC**	**Summary of Product Characteristics (in Dutch: officiële productinfomatie IB1-tekst)**
**Sponsor**	**The sponsor is the party that commissions the organization or performance of the research, for example a pharmaceutical company, academic hospital, scientific organization or investigator. A party that provides funding for a study but does not commission it is not regarded as the sponsor, but referred to as a subsidizing party.**
**SUSAR**	**Suspected Unexpected Serious Adverse Reaction**
**TUM**	**Technical University of Munich**
**TBR**	**Tumor-to-Background Ratio**
**UMCG**	**University Medical Center Groningen**
**UAB**	**University of Alabama at Birmingham, Birmingham, USA**
**VEGF**	**Vascular Endothelial Growth Factor**
**Wbp**	**Personal Data Protection Act (in Dutch: Wet Bescherming Persoonsgevens)**
**WMO**	**Medical Research Involving Human Subjects Act (in Dutch: Wet Medisch-wetenschappelijk Onderzoek met Mensen**

Summary

Background

Surgery remains a main pillar in the treatment of head and neck squamous cell carcinoma (HNSCC). The margin status is the main prognostic factor of local tumor control in surgically treated HNSCC and will determine the postoperative treatment strategy. A margin of ≤1 mm of normal tissue is considered a positive margin and requires either a re-operation or postoperative chemoradiation with a combination of cisplatin and 5-FU, which substantially increases morbidity. Margins wider than 1 mm but less than 5 mm require re-operation, or, if that is not possible, post-operative radiotherapy without the concomitant use of chemotherapy. Currently, no technology is available in the operating room, which reliably supports tumor excision in terms of margin status. In fact, surgeons can only combine pre-operative imaging data with tactile and visual information during surgery for assessing tumor margins with limited accuracy. With the introduction of molecular imaging techniques using near infrared (NIR) fluorescent optical contrast agents coupled to targeted compounds, new avenues have opened up for intra-operative assessment of tumor margins. Tracers are based on antibodies directed against Vascular Endothelial Growth Factor-A, i.e. bevacizumab-IRDye800CW, in patients with breast cancer or against Epidermal Growth Factor Receptor, i.e. cetuximab-IRDye800CW, in patients with HNSCC. First trials have shown that systemic administration of these compounds is safe and tumor specific. These findings prompted us to design this innovative application in a clinical trial for the intraoperative assessment of tumor margins during surgical treatment of HNSCC using cetuximab-IRDye800CW. The study is subsidized by the Dutch Cancer Foundation.

Objectives

The main purpose is to establish the intraoperative use of cetuximab-IRDye800CW as a reliable marker for residual tumor in resection margins after surgical removal of HNSCC. The objective is to establish the positive predictive value of cetuximab-IRDye800CW fluorescence as a marker for a tumor positive resection margin.

Study design

The study is designed as a phase 1-2, single center prospective cross sectional diagnostic study in patients with HNSCC that require surgical excision. First, a dose finding study will be performed in 9 patients using 10, 25 and 50 mg of cetuximab-IRDye800CW with three patients per dose cohort. In the first and only performed study at the University of Alabama (UAB) using cetuximab-IRDye800CW in the visualization of HNSCC, the dose found to be

optimal was 25mg/m2. We therefore think that a sufficient dose will be found within the proposed range. The most optimal dose from the three studied doses will be used in the second part of the study which will include a cohort of 70 patients. The choice of cetuximab-IRDye800CW dose will be a balance between the lowest dose vs. a clinically usable tumor to background ration (TBR) on the fluorescence images.

During the second phase of the study tumor margins will be studied in a cohort of 70 patients to determine the positive predictive value of optical imaging to identify positive margins. Based on historical data retrieved from our HNSCC database at UMCG we anticipate in a cohort of 70 patients at least 14 (20%) margin-positive patients and a 90% EGFR overexpression rate. We anticipate a sensitivity of 90% of the cetuximab-IRDye800CW conjugate based on the EGFR overexpression rate, which we will be able to measure with sufficient precision (95%CI of 60-96%).

Study population

Patients eligible for inclusion should suffer from a squamous cell carcinoma in the head and neck region (HNSCC) of which the head and neck tumor board of the UMCG has advised to be treated by surgical removal. Detailed description of inclusion and exclusion criteria are given in paragraph 2.3.

Patient related study procedures

Tracer administration: patients will visit the hospital four days prior to the planned surgery of their HNSCC. The cetuximab-IRDye800CW will be injected by slow infusion and patients will be monitored for potential side effects. The dose will be either 10, 25 or 50 mg of cetuximab-IRDye800CW which is less or equal to10% of the dose of cetuximab when used for curative treatment of HNSCC (usually 400mg/m2 loading dose and 250mg/m2 maintenance dose).

Intraoperative fluorescence imaging and spectroscopy

Study aims: The aim is to identify squamous cell carcinoma as fluorescent spots in the margin of a tumor resection specimen or in the wound bed in the patient.

Parameters: Fluorescence imaging and spectroscopy: Fluorescence images will provide an overview of where cetuximab-IRDye800CW fluorescence is located in the resection specimen and the wound bed in the patients. The intra operative camera is very sensitive for cetuximab-IRDye800CW fluorescence. One drawback is that on the fluorescence image the exact depth from which the fluorescence signal is generated cannot be established.

Furthermore, most likely there will be background fluorescence signals from normal tissue. Therefore, confirmation of the fluorescence signal on images requires quantification of the fluorescence signal. This can be performed by using a spectroscopy technique (MDSFR spectroscopy) that can quantify (in M/m^3) specifically IRDye800CW-fluorescence by placing a fiber tip in contact with the tissue. This spectroscopy technique has a shallow sampling depth of 1-2 mm. If fluorescence is generated from deeper layers, the signal of spectroscopy will be low (only background signal from muscle, connective tissue and salivary glands). If the IRDye800CW-fluorescence signal is generated from tumor in the resection margin the fluorescence signal will be much higher because SCC-tumor cells overexpress EGFR. The parameter that will be established is the threshold level at which background cetuximab-IRDye800CW spectroscopy signal can be separated from much higher spectroscopy signals of cetuximab-IRDye800CW accumulated in tumor.

Pathology: The tumor specimen will be processed for histology according to the current standard used in clinical cancer care. Diagnosis on margins, selected histological features necessary for clinical decision making will be provided. Next to this fluorescence images will be collected from the tumor specimen and biopsies. Margin width and number of positive margins will be noted and correlated to the location of fluorescent locations in the margins. From this positive predictive value will be calculated.

Burden, risks and benefit related to participation

Burden - Time investment: Patients need to make one extra visit to the UMCG four days before their planned surgery that will take approximately 2 hours. Usually patients are admitted one day prior to the planned surgery. Therefore the measurements one day before surgery will not require extra time investment

Burden-extra procedures: **1)** Intravenous administration of cetuximab-IRDye800CW. **2)** Fluorescence images will be taken from the tumor one day prior to surgery in the first cohort of nine patients. **3)** The estimated time for taking fluorescence images and spectroscopy measurements is approximately 30min. Therefore the time under general anesthesia will be prolonged. The usual time of surgical procedures for removal of HNSCC ranges from 2 hours to 15 hours, depending on complexity of the surgical procedure. **4)** from the wound bed in the patient that exists after tumor excision, biopsies will be taken in the ongoing general anesthesia, of spots positive of cetuximab-IRDye800CW as seen on the fluorescence imaging and confirmed by spectroscopy.

Risks: Allergic reactions to cetuximab have been reported but this is considered a low risk. No preclinical or clinical study reported higher than grade 2 adverse events. the first study with cetuximab-IRDye800CW no serious events were reported in six patients.
Benefit: Patients will have no benefit from this study directly. Surgery will be planned as usual. During surgery, no decisions will be made based on the fluorescence imaging. The benefit of this study will be the establishment of usefulness of cetuximab-IRDye800CW during surgery to identify margins containing tumors. The results of these types of study will be at least beneficial for other patients with cancer in the future. Clinical experience will be obtained with fluorescent labeled antibody in intra operative margin assessment during surgery of HNSCC.

1. INTRODUCTION AND RATIONALE

1.1 The importance of a free surgical margin in head and neck squamous cell carcinoma

The Dutch guidelines state that the preferred treatment of Oral Squamous Cell Carcinoma (OSCC) and specific other operable head and neck SCC (HNSCC) is surgery followed by (chemo)radiotherapy when tumor margins are close.(1) The most important risk factor for local recurrence after surgery is margin status.(2-4) Most authors agree that surgical resection should aim to obtain at least a 5 mm margin on histopathological slides. (5,6) When the surrounding normal tissue is between 1-5 mm thick it is referred to as a close margin. A positive margin shows tumor cells within 1 mm of the surgical margin. This leads to a worse prognosis compared to tumor removal with negative margins, even after postoperative (chemo)radiation. (2,7,8)

A group of 272 OSCC patients that underwent surgery at the UMCG Department of Oral and Maxillofacial Surgery was retrospectively analyzed. 20% (54/272) of the excised tumors showed positive margins (1 mm or less) over all four T stages (Table 1). These results are in concordance with the literature (6,9) According to the current treatment standards, in the case of positive margins that cannot be re-resected, patients under 70 years and in good condition undergo post-operative chemoradiation. This induces a higher morbidity and results in poorer dietary intake due to reduced swallowing capability and late fibrosis. (2,10) Prevention of postoperative chemoradiation by obtaining sufficiently wide surgical margins improves the quality of life of treated patients. (11)

Margin status	T1	T2	T3	T4	Number	%
**Clear**	77	56	2	19	154	57
**Dysplasia**	9	2	0	2	13	4
**Close**	20	16	6	9	51	19
**Positive**	**17**	**15**	**5**	**17**	**54**	**20**
**Total**					272	100

Table 1: **Summary of retrospective analysis of tumor margin status after surgical resection of OSCC.**

1.2 The rationale of intraoperative Near Infrared Fluorescent imaging in surgical removal of HNSCC

When addressing the issue of positive margins, an important distinction should be made between mucosal margins and deep connective tissue margins. Surgeons can use

intraoperative frozen sections to assess the superficial epithelial margin. The pathologist rapidly analyses a snap frozen section of the epithelial margin for the presence of tumor. This provides the surgeon with the opportunity to increase the resection margin. The survival of patients with a negative epithelial margin on frozen sections was similar when compared to patients with a positive epithelial margin that was converted to a negative margin by re-resection. (12) Therefore, immediate re-resection in cases with a positive mucosal margin is considered an adequate strategy to reduce the number of recurrences.

Surgeons are more likely to encounter difficulties in achieving adequate margins for deep connective tissue planes. (6) 87% of the positive surgical margins are located in the deep planes, while only 16% is located at the epithelial surface. (7) Ultrasound showed limited capabilities for assessing the deep tumor margin. (13) More recent DNA techniques analyzing for specific genetic or methylation alterations in surgical margins have not yet proven their usefulness. (14) In conclusion, there is no technique available for intraoperative assessment of deep connective tissue margins during surgical removal of HNSCC. The application of near infrared intraoperative imaging in HNSCC may help in the identification of deep connective tissue margins.

When histopathological evaluation shows that a margin is positive, adjuvant treatment is necessary. In cases of small tumors (T1) or a superficially located positive margin a re-resection can be performed. However, often (87% of the cases) the margin is positive at a deeper seated margin in the deeper connective tissue layers. Usually it is not possible to exactly locate the region of the positive margin and the choice for adjuvant therapy than will be post-operative concomitant chemoradiation. Therefor a method that will aid the surgeon to identify during surgery where the margin is close could be of great benefit for the patient and reduce the morbidity of the post-operative treatment.

1.3 Intraoperative near infrared fluorescence imaging in cancer

Until recently, the application intraoperative near infrared fluorescent imaging was limited due to a lack of specificity of fluorescent probes for tumor tissues. The first in vivo study to show the full potential of optical molecular imaging was performed at the University Medical Center Groningen (UMCG) using a folate-FITC (fluorescein isothiocyanate) conjugate for the detection of intra-abdominal metastasis from ovarian cancer. (15) The contrast agent showed a high specificity for tumor cells with upregulated FR-α receptors. Furthermore, images with a good tumor-to-background ratio (TBR) could be obtained. The maximum absorption of FITC is centered on 495 nm. A disadvantage of this wavelength is the difficulty to separate the

probe fluorescence from both tissue autofluorescence (induced by connective tissue and keratin). Also, differences in the optical properties of tissues influence fluorescence light transport. The introduction of compounds with absorption and emission spectra in the Near Infrared (NIR) region opened up the field of image-guided surgery. For many years indocyanine green (ICG) was the only NIR dye used for optical diagnostics, mainly for angiography and the visualization of tissue perfusion. (16) ICG has been used as a fluorescent probe for sentinel lymph node biopsy in various types of cancer, including HNSCC. (17,18) The use of ICG conjugated monoclonal antibodies (mAbs) has been limited, because the quantum yield of fluorescence of ICG is reduced when it is covalently bound to a protein. (16) With the introduction of IRDye800CW, (suitable for conjugation with mAbs) molecular-specific fluorescence for image-guided surgery became clinically applicable. (19)

In recent years, significant progress is made in the development of optical imaging devices for intraoperative near infrared fluorescence (NIRF) imaging. In collaboration with the Technical University of Munich (TUM), a real-time optical imaging system has been developed using video-rate concurrent multi-spectral near infrared and visible light imaging at a molecular level. (20,21) Following the intravenous administration of an fluorescent tracer, an external light source with a defined wavelength is used to illuminate the subject. As light propagates through the tissue, it will excite both surface and subsurface localized optical contrast agents (22). Immediately after excitation, the contrast agent responds by releasing low-energy light of a longer wavelength, which can subsequently be detected by a highly sensitive charge-coupled (CCD) camera. More recently, the company SurgVision improved the initial TUM setup by introducing the SurgVision F2 multispectral imaging system. This updated system is currently in use at the UMCG. Improvements include upgrades of crucial white light elements, white light camera, the CCD and optical filter sets. This resulted in a higher and more efficient signal excitation and therefore better fluorescent signal visualization. The setup was further improved by adding connectors for fiber optic devices, which made a connection with endoscopic devices possible. This allows for a more easy access of anatomically deeper situated areas such as the esophagus, colon and the oral cavity for NIRF imaging.

1.4 The added value of spectroscopy in clinical decision making during fluorescence image-guided surgery

A number of fundamental research questions remain regarding the use of optical contrast agents in image-guided surgery. An important issue is that imaged fluorescence is strongly affected by the optical properties of the tissue (defined by the absorption and scattering

coefficient). (23) These optical properties vary dramatically between different regions of interest within a wide-field optical image. The interpretation of signals uncorrected (or not fully corrected) for the tissue optical properties is extremely challenging. Separating true localization of a fluorescent probe from scattered signals is possible by quantification of the signal, which is not (yet) possible with intraoperative fluorescence imaging. Separation of IRDye800CW fluorescence from background autofluorescence is not possible using wide field imaging. Even with the choice of IRDye800CW, which shows absorption in the region where autofluorescence is low (i.e. the diagnostic window), identification of the fluorescence signal is necessary for clinical decision making. Also free blood in the imaging field has the potential to confound the use of optical imaging. Therefore, new methods to determine the presence of blood warranted. Knowledge of the photon path-length is necessary to quantify the optical properties of tissue.

A possible solution might be to use an optical technique in which the path-length of light can be controlled. An example of such a technique is single fiber reflectance (MDSFR) spectroscopy, which was recently developed at the Erasmus MC. (24-32) In MDSFR white light is emitted through a single fiber that is in contact with the tissue. The same fiber collects the reflected light and can be used to acquire a reflectance spectrum over a broad range of visible and NIR wavelengths. This reflectance spectrum contains the combined information on how much light has been absorbed and scattered. From such a reflectance spectrum the tissue absorption coefficient, μa [mm-1] can be accurately quantified without prior knowledge of the scattering properties. (26) To determine the tissue scattering properties, however, a single MDSFR measurement is insufficient. The optical geometry in MDSFR allows superficial measurements with relatively short photon path-lengths. As a result, the number of scattering events is relatively small. This means that the angle in which the light is scattered becomes increasingly important for the amount of reflected light. This “scattering angle” follows a probability distribution that is described by the (unknown) tissue phase function. Because the reflectance in MDSFR is highly dependent on this phase function it is necessary to characterize it in order to measure the reduced scattering coefficient μs’[mm-1].

1.5 In vivo NIRF imaging: Clinical trials performed at the UMCG

The first in-human application of intraoperative tumor-specific fluorescence imaging using folate-FITC in patients with ovarian cancer was performed in the UMCG, resulted in an extensive experience with in vivo fluorescence imaging of solid tumors using targeted fluorescent probes. Ongoing clinical trials focus mainly on bevacizumab-800CW. These trials include NIRF imaging of gynecological tumors, breast cancer, esophageal cancer and

colorectal cancer (Table 2). Preliminary data from breast cancer imaging (NCT01508572; NCT02583568) indicate that fluorescence of superficially located tumors can be visualized relatively easy (Figure 1). First results from our trial in patients with familial adenomatous polyps (NCT02113202) show that bevacizumab-800CW NIRF imaging is also feasible using a NIRF endoscopy platform, developed by our collaborators at the Technical University of Munich/Helmholtz (Figure 2). While the full analysis of the data acquired in the ongoing clinical trials is still underway, the potential of using bevacizumab-800CW for tumor specific molecular imaging is clear.

Indication	Subjects planned	Total dose (mg)	Status	Subjects enrolled	Reference
**Breast cancer study I**	20	4.5	Completed	20	NCT01508572
**Peritoneal carcinomatosis**	10	4.5	Enrolling	7	NL45588
**Colorectal cancer***	30	4.5	Enrolling	22	NCT01972373
**Malignant esophageal lesions***	10	4.5	Completed	10	NCT02129933
**Familial adenomatous polyps**	15	4.5, 10, 25, 50	Completed	15	NCT02113202
**Breast cancer II**	26	4.5, 10, 25, 50	Enrolling	4	NCT02583568

Table 2 **Overview of the ongoing and completed clinical trials using bevacizumab-800CW.**

*Endoscopy study

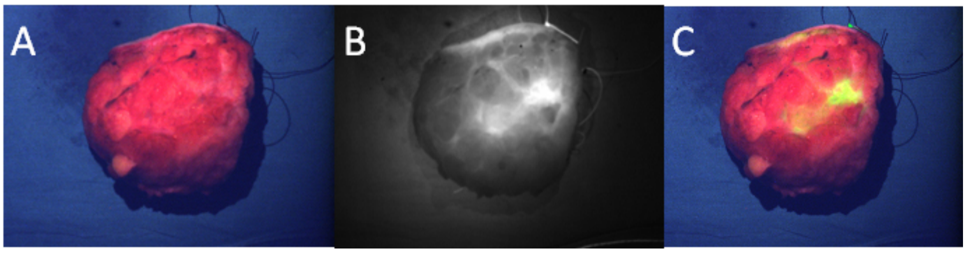

Figure 1 **Ex vivo images of excised breast cancer with the macroscopic view** (A), fluorescence image of bevacizumab-IRDye800CW (B)and the overlay of macroscopic view and fluorescence image (C).

1.6 Rationale for the use of cetuximab-IRDye800CW as fluorescent tracer in HNSCC

Multiple agents are currently being applied for targeted therapy in HNSCC. These include bevacizumab, cetuximab and lapatinib. (33) Bevacizumab is a monoclonal antibody that binds to vascular endothelial growth factor (VEGF). A disadvantage of VEGF is that it is overexpressed in both HNSCC and peritumoral vasculature and lymph beds. The higher background signals of these tissues make bevacizumab less attractive for fluorescence imaging. (34) Recently, we imaged solid tumors in animals using bevacizumab and

trastuzumab (a MAb targeting HER2). A significantly lower TBR of was found for evacizumab when compared to trastuzumab. (35) Cetuximab is a monoclonal antibody that targets epidermal growth factor receptor (EGFR). It was approved for HNSCC treatment in 2006. (36) EGFR is significantly overexpressed in more than 90% of HNSCC.(35,37) ^{89}Zirconium-cetuximab showed good localization in HNSCC in PET imaging. (38) Recently, our collaborators at the Department of Otolaryngology and Head and Neck Surgery of the University of Alabama at Birmingham (UAB) published the first data from patients with HNSCC injected with cetuximab-IRDye800CW (ClinicalTrials.gov, NCT01987375) (39). The localization of fluorescently labeled cetuximab and the relation to the tumor margin was assessed. Tumors were imaged using a real-time NIRF imaging system (NOVADAQ). The surgical pathology specimens of both tumor and normal tissues were imaged using a closed field imaging system (Odyssey) prior to histological sectioning.

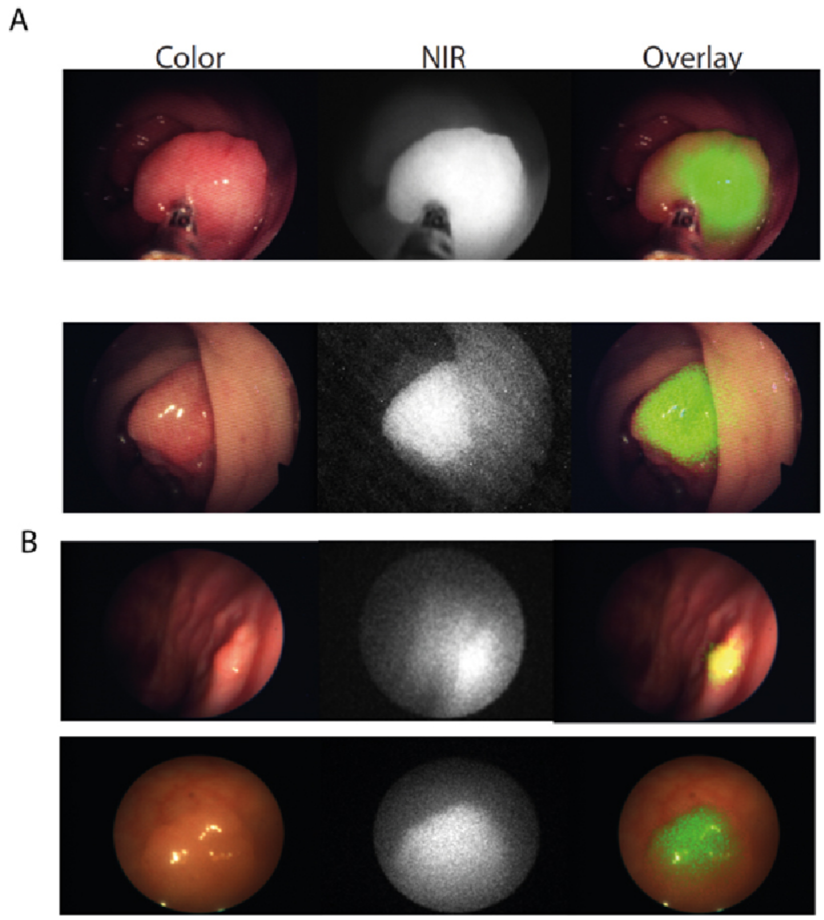

Figure 2 **Preliminary results of clinical molecular targeted NIR endoscopy using bevacizumab-IRDye800CW.** White light and NIR fluorescence endoscopy with bevacizumab-IRDye800CW demonstrates to be highly specific with high uptake of the tracer in tumor, low uptake in normal tissue in two patients (A) and between adenomas and normal tissue in another two patients (B).

The first in vivo images in superficial tumors surrounded by skin, showed that cetuximab-IRDye800CW fluorescence can be imaged in HNSCC with a good TBR (Figure 3). The TBR of cetuximab-IRDye800CW was 2.2, 2.5, and 1.9 at respectively 2.5 mg/m^2, 25mg/m^2 and 62.5 mg/m^2 doses. When measuring with fluorescence microscopy, the mean fluorescence intensity (MFI) of just the tumor, a 10-fold increase in single infusion dose (25 mg/m^2 vs. 2.5 mg/m^2) resulted in an almost 7-fold increase in MFI. Yet, further 25-fold increase in infusion dose (2.5 mg/m^2 vs. 62.5 mg/m^2) yielded only 8.8-fold increase in the MFI. Apparently there seems to be a tradeoff between the tumor uptake and an increasing background fluorescence at increasing doses. A clear demarcation in fluorescence intensity exists between tumor and normal tissue with low fluorescence signals in normal tissue, see figure 4.

A poor correlation between cetuximab-IRDye800CW fluorescence and EGFR expression was only found in areas of mature, differentiated keratinizing cancer or areas of tumor necrosis. Cetuximab-IRDye800CW fluorescence outside the tumor was observed on the cell membrane of the basal cells and in certain areas of sebaceous glands. No grade 2 or higher toxicity events have been reported (For detailed information about safety and toxicity of cetuximab-IRDye800CW, see section 6.4).
These results indicate that the application of cetuximab-IRDye800CW for intraoperative fluorescence imaging is feasible and safe. This prompted us to aim for the clinical translation of cetuximab-IRDye800CW for intraoperative application in HNSCC. The exact demarcation

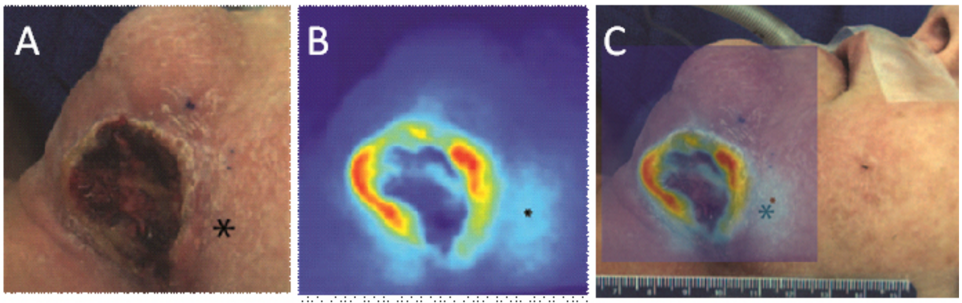

Figure 3 **Intraoperative images of cetuximab-IRDye800CW in HNSCC**
Macroscopic (A), fluorescence (B) and overlay of A en B (C) The patient in the images had an eroding diseased lymph node in the neck. The origin was SCC of the lip. She received composite resection with neck dissection. At 3 days post cetuximab-IRDye800CW infusion (25mg/m^2), there was a TBR of 5 observed during open-air wide field fluorescence imaging of the lesion. The cetuximab-IRDye800CW is clearly visible and usable for identification of the tumor. On the right side of the tumor, marked by an asterisk (*), cetuximab-IRDye800CW fluorescence in a subdermal part of the SCC is visible through the overlying skin. The central part of the tumor is necrotic and shows low uptake of the conjugate, as was confirmed by fluorescence microscopy (not shown). The exact border of the tumor is difficult to establish due to scattering of the fluorescence signal, which can be overcome by quantifying the signal with spectroscopy.

of tumor margins in study performed at the UAB was difficult due to scattering of the fluorescence signal. The application of quantitative spectroscopy as proposed in our study might improve the accuracy of the intraoperative identification of tumor margins.

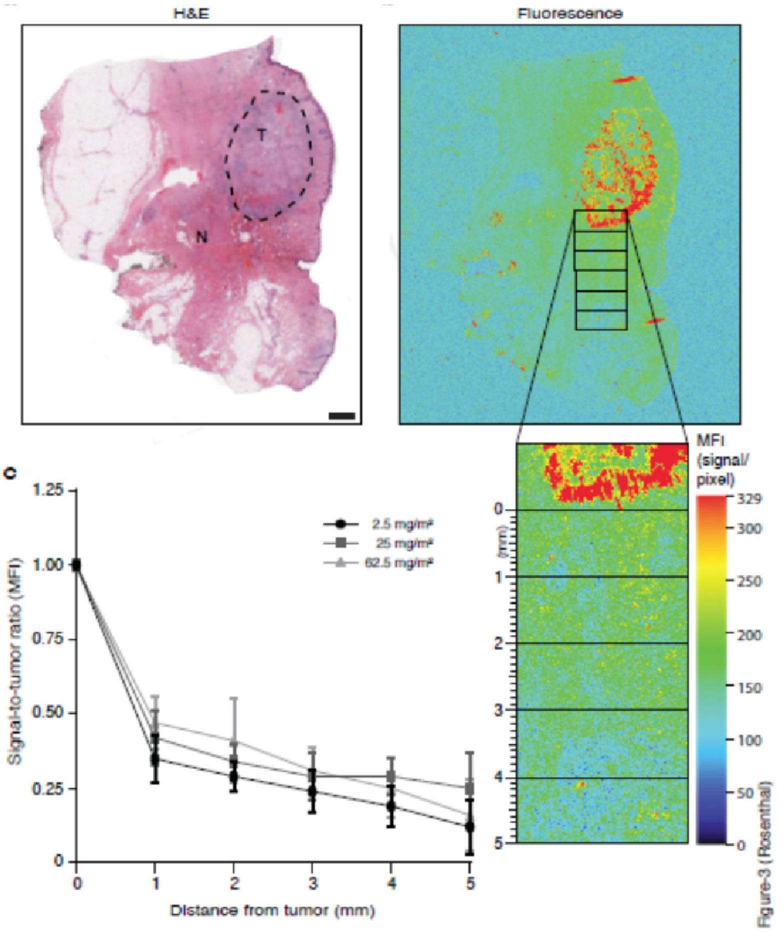

Figure 4 **Microscopy of HNSCC injected with cetuximab-IRDye800CW three days prior to surgery.** Fluorescence from adjacent normal tissue correlates with distance from tumor. Representative HE image tumor (T) and normal (N) regions(A). Mean fluorescence intensity (MFI) is determined at predefined distances from tumor (1-5 mm) as depicted by rectangular regions-of-interest (ROI) at 0, 1, 2, 3, 4 and 5 mm margin distance (B) graphed (C). Data are presented as mean ±SD. Scale bar represent 50 µm. As can been seen in this image that low fluorescence signals are present in normal tissue and thus finding the threshold level between normal EGFR distribution and tumor related EGFR seems possible.

1.7 Dose selection for the cetuximab-IRDye800CW conjugate

The doses of 2.5, 25 and 62.5 mg/m^2 cetuximab-IRDye800CW as administered in the recently performed phase-1 dose escalation study at the UAB equal doses of 4, 42 and 106 mg assuming a body surface of 7.71 m^2 (39). Comparison of the NOVADAQ (used at the UAB) and the SurgVision F2 (used at the UMCG) multispectral imaging systems showed that the SurgVision system is approximately 40 times more sensitive (attachment K6.6). We

therefore decided to perform a dose finding study of cetuximab-IRDye800CW in HNSCC in our setting using the SurgVision F2 multispectral imaging system with an attached nasopharyngeal endoscope (see section 7 for detailed information of devices used in this study) to identify the most optimal dose of cetuximab-IRDye800CW for our imaging system. The dose of 2.5 mg/m^2 as administered in the dose escalation study can be considered microdosing and is comparable to the dose of 4.5 mg used in ongoing UMCG studies using bevacizumab-800CW. From these studies we have learned that a dose of 4.5 mg bevacizumab-IRDye800CW is too low to reliably measure a fluorescence signal or perform adequate spectroscopy (personal communication dr. Nagengast (UMCG) and dr. Robinson (Erasmus Medical Center)). Furthermore, the dose escalation study performed at the UAB reported TBR levelling with higher cetuximab-IRDye800CW doses (See section 1.6). Also because of the higher sensitivity of our system as found by calibrating both devices and the TBR levelling with higher cetuximab-IRDye800CW doses, we hypothesized that a lower maximum dose than administered at the UAB may be sufficient for an adequate TBR in our setting. The administrated doses of cetuximab (10, 25 and 50mg) as proposed for this study are regarded safe (see section 6.4) and correspond to a fraction of the therapeutic dose (400mg/m^2 loading dose and 250mg/m^2 maintenance dose) which is given during concurrent chemoradiation. For these reasons we think it is appropriate to select doses of 10, 25 and 50mg of cetuximab-IRDye800CW and test each dose in a cohort of 3 patients.

2. OBJECTIVES

2.1 Primary objective Part 1 (dose finding)

- To determine the optimal dose of cetuximab-IRDye800CW for intra operative imaging with the SurgVision F2 multispectral imaging system for easily accessible anatomical areas in the head and neck region, or with a standard nasopharyngeal endoscope attached to the SurgVision F2 multispectral imaging system for difficult accessible anatomical areas in the head and neck region.

2.2 Primary objective Part 2 (main study)

- The main purpose is to establish the intraoperative use of cetuximab-IRDye800CW as a reliable marker for residual tumor in resection margins after surgical removal of HNSCC. The objective is to establish the positive predictive value of cetuximab-IRDye800CW fluorescence as a marker for a tumor positive resection margin.

2.3 Secondary Objectives

- To determine the threshold level ***in vivo*** of cetuximab-IRDye800CW fluorescence for reliable intraoperative deep margins assessment with high sensitivity while ensuring an adequate positive predictive value.
- To quantify sensitivity and positive predictive value of cetuximab-IRDye800CW fluorescence of HNSCC ***ex vivo*** using optical molecular imaging and MDSFR/SFF versus fluorescence microscopy and EGFR immunohistochemistry.
- To obtain information on safety aspects of cetuximab-IRDye800CW administration by registration of conjugate blood levels, conjugate integrity, side effects, adverse events (AE), serious adverse events (SAE) and suspected unexpected serious adverse reactions (SUSAR).

2.4 End points Part 1 (dose finding)

The primary endpoint of the dose finding study is to identify a dose corresponding to a sufficient Tumor to Background Ratio (TBR). The TBR will be calculated by a combined assessment of intraoperative *in vivo* and *ex vivo* fluorescent signals (SurgVision F2 multispectral imaging system, nasopharyngeal endoscope, MDSFR/SFF spectroscopy) together with *ex vivo* examinations (histological examination, NIR flatbed scanning and fluorescence microscopy).

Secondary endpoints will be:

1) Safety data (cetuximab-IRDye800CW blood levels and conjugate integrity, side effects, AE, SAE, SUSAR);
2) Localizing patterns of cetuximab-IRDye800CW fluorescence within both tumor and normal tissue by fluorescence microscopy and histological examination.

2.5 End points Part 2 (main study)

The primary endpoint to calculate the sensitivity, specificity and positive predictive value of cetuximab-IRDye800CW-fluorescence of tumor margins by a combination of intraoperative *in vivo* and *ex vivo* assessment of the of the surgical wound bed following excision of the primary tumor (SurgVision F2 multispectral imaging system, nasopharyngeal endoscope, MDSFR/SFF) and *ex vivo* examinations of the acquired biopsies (histological examination, NIR flatbed scanning and fluorescence microscopy)

Secondary endpoints will be:

1) Safety data (cetuximab-IRDye800CW blood levels and conjugate integrity, side effects, AE, SAE, SUSAR);
2) Assessment of the minimal thickness of the non-fluorescent margin of the excised specimen by intraoperative *in vivo* and *ex vivo* assessment (SurgVision F2 multispectral imaging system, nasopharyngeal endoscope, MDSFR/SFF) combined with *ex vivo* examinations (histological examination, NIR flatbed scanning and fluorescence microscopy)

3. STUDY DESIGN

The current study is a non-randomized, non-blinded, prospective, single center, cross-sectional diagnostic study. A maximum of 88 patients with HNSCC will be included. All included patients will meet with the in- and exclusion criteria and have a biopsy confirmed diagnosis of primary or recurrent HNSCC and are scheduled to undergo surgical resection as decided by the Multi-Disciplinary Head and Neck Tumor Board of the UMCG (see section 4. Study population). The intraoperative imaging of cetuximab-IRDye800CW will be performed using a combination of the “open air” SurgVision F2 multispectral imaging system (SurgVision BV, ‘t Harde, the Netherlands), a nasopharyngeal endoscope and MDSFR/SFF probe. A detailed description of the safety aspects, application and specifications of these devices can be found in section 7. A detailed description of the imaging protocol can be found in section 8.

The study consists of two parts. In part 1, the optimal cetuximab-IRDye800CW for intraoperative imaging will be determined. In part 2, determine the threshold level of cetuximab-IRDye800CW fluorescence for reliable intraoperative deep margins assessment with high sensitivity while ensuring an adequate positive predictive value will be determined. A detailed description of the rationale of the imaging protocol can be found in section 3.3.

3.1 Study design Part 1 (dose finding)

Part 1 of the study will consist of 3 study cohorts with a maximum of 3 patients for each cohort. The doses for each consecutive cohort will increase from 10, 25 to ultimately 50 mg cetuximab-IRDye800CW. The administered doses of cetuximab are expected to be safe and are significantly lower than doses as administered in a therapeutic setting (Section 6.4 and see IMPD section 2.1.S.1.3) A detailed justification of the doses as proposed for this dose finding study is described in section 1.7 We estimate that three patients for each dose is sufficient for adequate TBR calculation. For each case the TBR will be calculated as described in section 8.1. Our aim is to confirm whether these prior results apply to our

setting. Depending on the outcome of the DSMB review in terms of (S-)AEs we will continue with part 2 of our study. If necessary, the DSMB may advise to increase each dose cohort to 6 patients (3+3), thereby increasing the maximum number of patients included in part 1 of the study to 18 (Figure 5).

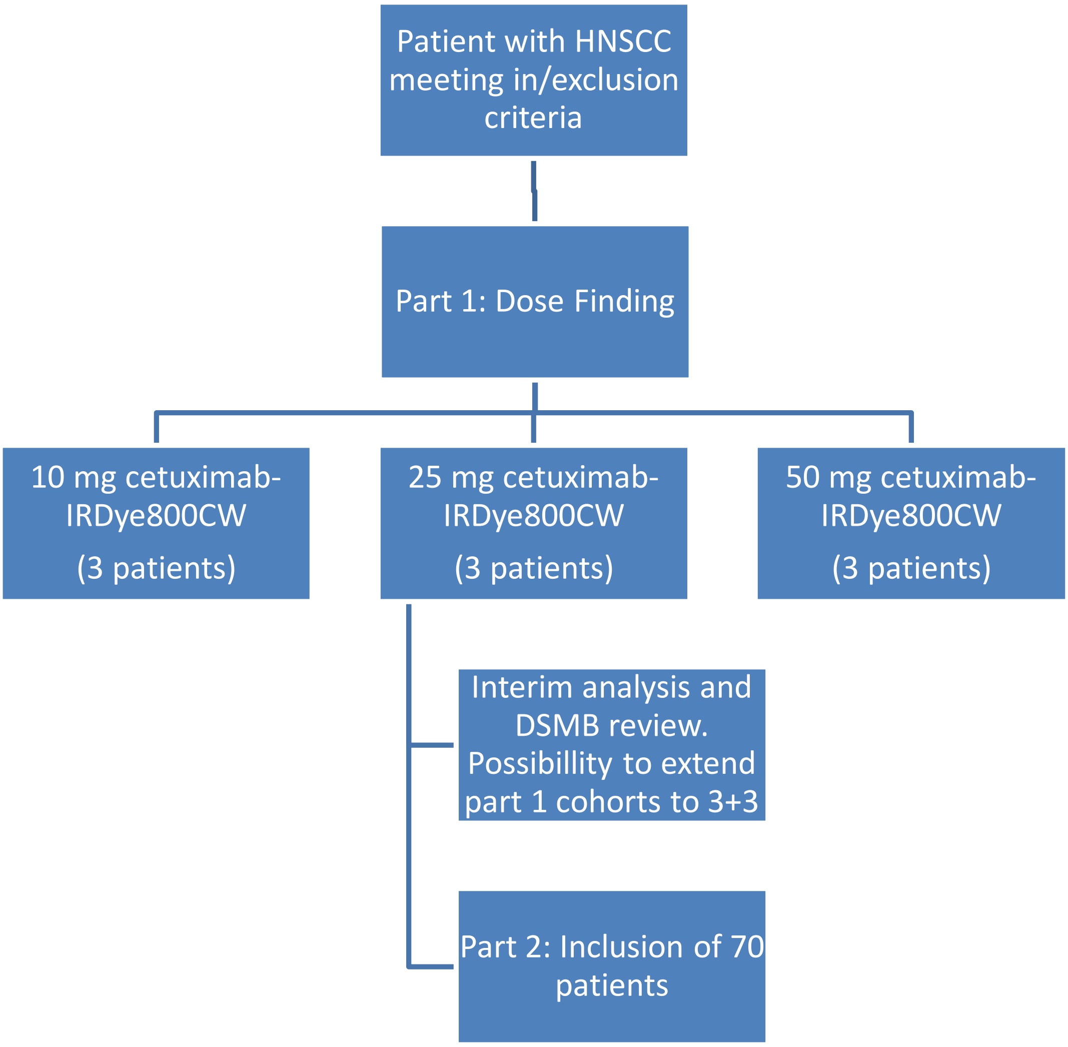

Figure 5 **Study Design Flow Chart**

3.2 Study design part 2

Part 2 of this study will consist of 70 patients. The administered dose is based on the optimal cetuximab-IRDye800CW dose as identified in part 1 of our study during the dose finding. The rationale of this sample size calculation can be found in section 4.4 "Sample size calculation." An interim analysis will be performed following the inclusion of 35 patients. Depending on the outcome of the DSMB review in terms of (S-)AEs we will resume with inclusion of the final cohort of 35 patients (Figure 5).

4. STUDY POPULATION

4.1 Population (base)

Patients with primary or recurrent HNSCC, who are scheduled to undergo surgical resection as decided by the Multi-Disciplinary Head and Neck Tumor Board of the UMCG.

4.2 Inclusion criteria

1) Biopsy confirmed diagnosis of primary or recurrent HNSCC and scheduled to undergo surgical resection as decided by the Multi-Disciplinary Head and Neck Tumor Board of the UMCG.
2) Age ≥ 18 years
3) Written informed consent
4) Adequate potential for follow up
5) Acceptable hematologic status, kidney function, and liver function, as standard surgery protocol requires.

4.3 Exclusion criteria

1) Medical or psychiatric conditions that compromise the patient's ability to give informed consent
2) Concurrent uncontrolled medical conditions.
3) Received an investigational drug within 30 days prior to the dose of cetuximab-IRDye800CW
4) Tumors at sites of which the surgeon would assess that in vivo imaging would not be feasible
5) Had within 6 months prior to enrollment: myocardial infarction, cerebrovascular accident, uncontrolled cardiac heart failure, significant liver disease, unstable angina
6) Inadequately controlled hypertension with or without current antihypertensive medications.
7) History of infusion reactions to cetuximab or other monoclonal antibody therapies
8) Pregnant or lactating women. Documentation of a negative pregnancy test must be available for women of childbearing potential. Woman of childbearing potential are premenopausal women with intact reproductive organs and women less than two years after menopause.

9) Evidence of QT prolongation on pretreatment ECG (greater than 440 ms in males or greater than 450 ms in females)
10) Lab values that in the opinion of the primary surgeon would prevent surgical resection.
11) Patients receiving Class IA (quinidine, procainamide) or Class III (dofetilide, amiodarone, sotalol) antiarrhythmic agents.
12) Magnesium, potassium and calcium lower than the lower limit of normal range.
13) Life expectancy < 12 weeks
14) Karnofsky performance status < 70%

4.4 Sample size calculation

Patients will be selected from the group of patients that will be surgically treated for HNSCC at the UMCG. The maximum number of patients included for this study will be 88 (Part 1: 9(+9) and Part 2: 70) if the DSMB advises to include more patients following the interim analysis in the dose finding study. The patients will be included within 4 years. We are confident that it is reasonable to include the required amount of patients within in the Head and Neck unit of the UMCG during this time frame. In 2015, more than 100 operations for removal of HNSCC were performed at the department of Oral and Maxillofacial Surgery of the UMCG.

4.4.1 Sample size calculation Part 1 (dose finding)

The primary aim of part 1 is to identify the optimal dose of cetuximab-IRDye800CW for intraoperative detection of HNSCC, defined as a sufficient TBR. The dose finding part involves a small sample size of 9 patients (3 cohorts with 3 patients each). Based on prior results from the UAB, we estimate that this sample size is sufficient to verify an optimal dose in our setting. An interim analysis will be performed following the inclusion of all 9 patients. This interim analysis will be discussed with the DSMB. If data is insufficient, a maximum of 9 additional patients will be included for analysis in the dose finding study.

4.4.2 Sample size calculation Part 2 (main study)

Part 2 will include 70 patients. We expect that 20% (n=14) of the included patients will have positive deep margins. As each patient may have more than one ROI with positive margins, and as fluorescence positive ROIs will be sampled already at a low signal, this will lead to sufficient data to evaluate the spectroscopy signal for discriminative value and derive an

optimal threshold (given the necessary 10 events per predictor). Furthermore, we will use bootstrapping methods to correct for optimism in threshold finding. With regard to the per-patient analysis, we expect that approximately 90% of patients with a positive margin will show EGFR overexpression in that margin and will thus be detected by the spectroscopy signal. If true, this will lead to 12 of 14 patients with positive margins to be detected. Given the sample size, this results in a sensitivity of 86% with a corresponding 95% C.I. of 60-96% (at 71% sensitivity, the 95% C.I. will be 45-88%, and at 50%: 27-73%), yielding sufficient precision as to the expected impact on margin status of imaging-guided surgery and thus allowing the informed design of a subsequent comparative randomized study. Estimates of specificity will be even more precise given the larger number of patients with negative margins.

5. TREATMENT OF SUBJECTS

5.1 Investigational product/treatment

The investigational product is cetuximab-IRDye800CW. This is in the literature also sometimes referred to as cetuximab-800CW or cetuximab-IRDye800. In this protocol it is also referred to as 'the tracer' or 'conjugate'. Cetuximab-IRDye800CW is administered intravenously 4 days before routinely scheduled surgery at multiple increasing doses (10, 25 and 50 mg) during part 1 of this study. The optimal dose of cetuximab-IRDye800CW as identified in part 1 of this study is administered intravenously 4 days before routinely scheduled surgery in 70 patients during part 2 of this study.

6. INVESTIGATIONAL PRODUCT

6.1 Name and description of investigational product(s)

The investigational medicinal product is an immune-fluophore conjugate composed of cetuximab (Erbitux®, Eli Lilly and Company, USA) and IRDye® 800CW (LI-COR, Inc. USA) with an average ratio of 1 molecule of cetuximab to 2 molecules of IRDye800CW. Cetuximab is a recombinant, human/mouse chimeric monoclonal antibody that binds specifically to the extracellular domain of the human epidermal growth factor receptor (EGFR). For general information regarding cetuximab is referred to the ‘summary of product characteristics’.
IRDye800CW is a near-infrared organic fluorophore IRDye800CW (800CW-NHS ester), produced under GMP conditions by REGIS Technologies, Inc.. For more information

regarding IRDye800CW is referred to the Active Substance Master File, Applicant's part AP/(v1) 2011-03-18 and Restricted part RP/(v1) 2011-03- 18.

Conjugation of the fluorescent dye to cetuximab, purification and formulation will be performed at the department of Hospital and Clinical Pharmacy of the UMCG. This process is described in the IMPD of cetuximab-IRDye800CW (IMPD version July 2016, section 2.1.s.2.2). In the literature, the conjugate is referred to as cetuximab-IRDye800CW or cetuximab-800CW. Both refer to the same product.

6.2 Summary of findings from non-clinical studies

See IMPD (version 1.0, July 2016), section 2.2, for a summary of findings from preclinical studies with cetuximab-IRDye800CW. For the summaries of preclinical studies on cetuximab, reference is made to the 'summary of product characteristics' of cetuximab, (Attachment D5-2, section 5.3)

6.3 Summary of findings from clinical studies

See IMPD (version 1.0, July 2016), section 2.3 for a summary of findings from clinical studies with bevacizumab-IRDye800CW. For the summaries of clinical studies on cetuximab, reference is made to the " summary of product characteristics" (SPC of cetuximab, section 5)

6.4 Summary of known and potential risks and benefits

The risks associated to the systemic exposure to the tracer are regarded minimal as the tracer mechanism of action is determined by the well described marketed medicinal product cetuximab (Erbitux®, see attachment D1 - SPC). The clinical safety profile of cetuximab is based on data from 5,500 patients with various malignancies including HNSCC, who received cetuximab at 6.75-10.8 mg/kg body weight. The initial dose for patients with metastatic HNSCC in combination with radiation- or chemotherapy is 10.8 mg/kg body weight, followed by 6.75 mg/kg once a week. This translates to a calculated plasma cetuximab of 150-450 mg cetuximab. Toxicity in with IRDye800CW at doses of 1, 5 and 20 mg/kg did not show any pathological effects. As no higher doses were tested the 20 mg/kg was identified as the no observed adverse effect level (NOAEL) (40) More recently, a toxicity study on cetuximab-IRDye800CW has been performed in macaques (Macada fascicularis) (39) The macaques were dosed at 250 mg/m2 (20.83 mg/kg), the full human therapeutic dose level of cetuximab. Cetuximab-IRDye800CW and cetuximab were tolerated well under

the conditions of the study, except for an increased QTc in both groups after dosing. The QTc returned to baseline levels at day 15 for the cetuximab control group, but not for the cetuximab-IRDye800CW group. Moreover, well described side-effects of cetuximab are hypomagnesemia, hypocalcemia and hypokalemia which can also cause a prolonged QT interval. For this reason, ECG and potassium, magnesium and calcium monitoring was included in the phase-1 dose escalation study of cetuximab-IRDye800CW (NCT01987375) (39). In this dose-escalation study, nine patients received 2.5, 25 and 62.5 mg/m^2 cetuximab-IRDye800CW intravenously. No grade 2 or higher toxicities related to the study drug where reported. Four possible related grade 1 adverse reactions occurred in the first cohort, four in the second cohort and two in the third cohort. This demonstrated that the toxicities were not dose related. The principal investigator of the study, prof. Rosenthal, concluded that these side-effects are most likely cetuximab related and not related to the conjugate. The QTc interval was significantly (P=0.028) higher at 2 hours compared to pre-infusion values, but returned to baseline at 30-days post-infusion.

6.5 Description and justification of route of administration and dosage

The tracer will be administered intravenously. This route of administration equals the established route of administration of cetuximab (Erbitux) which is justified as the mechanism of action of the tracer has been shown not to deviate from the mechanism of action of cetuximab (IMPD version 1.0, July 2016, section 2.2). A detailed justification for the doses selected in this study is provided in section 1.6 of this protocol and the IMPD version 1.0, July 2016, section 2.1.s.1.3).

6.6 Dosages, dosage modifications and method of administration

The tracer is administered by intravenous infusion. Cetuximab-IRDye800CW is provided as a ready to use solution (1mg/1ml). Preparation of a dose for use in the study consists of drawing the required volume of tracer into a syringe of sufficient size. No dilution steps will be performed on the product. Patient doses of 10, 25 or 50mg will be administered, therefore a syringe containing 10, 25 or 50ml will be prepared.

6.7 Preparation and labeling of Investigational Medicinal Product

All conjugates used in this study will be prepared, packaged and labeled under the responsibility of a pharmacist at the UMCG in accordance with the UMCG Standard Operating Procedures (SOPs), Good Manufacturing Practice (GMP) guidelines, International Conference on Harmonization for Good Clinical Practice (ICH-GCP) guidelines and applicable local laws and regulations. An UMCG qualified person will release the conjugate

in accordance with GMP guidelines annex 13 (Manufacture of investigational medicinal products) revision July 2010. The released trial medication will be stored in a secure area, inaccessible for unauthorized individuals at controlled temperature conditions of 2-8° Celsius. For a detailed description of preparation and labeling of the cetuximab-IRDye800CW, see IMPD (version 1.0 July 2016), section 2.1.p.

6.8 Drug accountability

There are no special requirements for the shipment, receipt, disposition, return and destruction of cetuximab-IRDye800CW.

7. Investigational medical device: Imaging devices

7.1 Name and description of non-investigational product(s)

The intraoperative imaging of cetuximab-IRDye800CW will be performed using a combination of the “open air” SurgVision F2 multispectral imaging system (SurgVision BV, ‘t Harde, the Netherlands), a nasopharyngeal endoscope (Diameter 3.5 mm, length 20 cm, Karl Storz GmbH & Co. KG, Tuttlingen, Germany) and MDSFR/SFF probes (CeramOptec GmbH, Bonn, Germany and LightGuideOptics GmbH, Rheinbach, Germany). The nasopharyngeal endoscope is a standard device and used in daily clinical practice in the UMCG. To substantiate the use of the SurgVision imaging system and MDSFR/SFF spectroscope, extensive risk analyses have been carried out (see section 7.5). The endoscope and MDSFR/SFF probe can be sterilized for intraoperative use (see attachment K6.4, K6.5.4 and K.6.6.1-2(MDSFR/SFF probe)).

7.1.1 SurgVision F2 multispectral imaging system

The multispectral F2 camera system provided by SurgVision is used for intraoperative NIRF imaging in this study (for user guide see attachments K6.1 and K6.1.1). The system is composed of two cameras, one camera with a light source in the visible spectrum and a fluorescence camera with a separate laser light source emitting at a wavelength of 750 nm. For specifications and acceptance report of the LED lighting source see attachments K6.1.2 and K6.1.3 respectively. The fluorescence camera is an Electron Multiplying Charge Coupled Device (EMCCD) camera enabling detection of only a few photons particularly under dynamic measurement conditions, providing a high sensitivity. The cameras are connected to a computer with customized software for processing of the images. One or more display screens are connected to the computer for the output signal. In the operating theatre the device will be covered with standard sterile drapes (i.e.: Zeiss OMPI no: 306071). As preparation for this protocol the imaging systems used at UAB and UMCG have been

compared to ensure that study outcomes will not be biased by major differences in equipment used. It was found that the UMCG used system is much more sensitive in detecting fluorescence than the system used at UAB (see attachment K6.6). Even when a standard nasopharyngeal endoscope was coupled to the SurgVision camera, this set-up outperformed the system used at UAB. Since the outcome measurements will be the TBR, which is a relative measure, the difference between the “open air” setting of the SurgVision multispectral imaging system and the nasopharyngeal endoscope will not influence the outcome of the study. The imaging system will be used to visualize the tracer and to guide the positioning of the MDSFR/SFF spectroscopy fibre. The MDSFR/SFF spectroscope will perform quantitative measurements at the fluorescent spots that will proved the data for this study to calculate the positive predictive value.

7.1.2 Flexible Nasopharyngeal endoscope

The “open air” SurgVision multispectral imaging system might not be able to image areas in the oral cavity or oropharynx due to anatomical restrictions. A standard flexible nasopharyngeal endoscope will be coupled to the SurgVision system to visualize the tumor and surgical wound bed following excision in these areas. Additional information about specifications and quality assurance of the nasopharyngeal endoscope is provided separately (attachment K6.3.1 and K6.3.2 respectively). The nasopharyngeal endoscope that will be used is a standard endoscope which is routinely used in the UMCG for inspection of the upper aerodigestive tract. When coupled to the imaging system it is possible to gain fluorescence images of sufficient quality and sensitivity. In attachment K6.6 the sensitivity of the SurgVison camera (open air or with the nasopharyngeal endoscope attached) compared to the system used at UAB for the first human trial of cetuximab-IRDye800CW. Even the endoscope the UMCG system is much more sensitive than the fluorescence imaging system from UAB.

7.1.3 MDSFR/SFF spectroscope

The principle device concerns a custom made Multi Diameter Single Fiber Reflectance/ Single Fiber Fluorescence (MDSFR/SFF) spectroscopy system which has been developed by the Erasmus MC in Rotterdam (Attachments K6.5, K 6.5.1, K6.5.2, K6.5.3 and K6.5.4). Two MDSFR/SFF probes will be used for both *in-vivo* and *ex-vivo* spectroscopy (Attachments K6.6, K6.6.1, K6.6.2). MDSFR spectroscopy and SFF spectroscopy; the former functioning to determine tissue absorption and scattering properties, the latter to detect

tissue fluorescence.(31,32,41) Illumination and detection are performed by a single optical fiber probe for both reflectance and fluorescence measurements. The measurement volume is confined to shallow depths of the order of the fiber diameter.(42,43) It has been shown that acquiring two successive single-fiber reflectance measurements with different fiber diameters (termed MDSFR) enables quantification of both the reduced scattering coefficient and the phase function parameter γ. This parameter is defined as= (1-g2)/(1-g1), where g1and g2 are the first and second Legendre moments of the phase function, and has to be included due to the shallow measurement geometry (29,32). Intrinsic fluorescence can be determined using a semi-empirical model based on Monte-Carlo simulations and phantom studies. This model incorporates the effect of optical properties on local excitation fluorescence and fluorescence photon escape probability within the whole sample volume.(29,41-44) The MDSFR/SFF spectroscope will perform quantitative measurements at the fluorescent spots that will proved the data for this study to calculate the positive predictive value. The MDSFR/SFF spectroscope is the same system that is used in other ongoing studies ('Intraoperative detection of cancer tissue in pancreatic adenocarcinoma using a VEGF-targeted optical fluorescent imaging tracer -A multicentre feasibility dose escalation study"- NL50488.042.15 en de VICE studie "Visualization of a VEGF-targeted Near-Infrared Fluorescent Tracer in patients with Familial Adenomatous Polyposis during Fluorescence Endoscopy" NL-45148.042.13). We have attached documents that were previously used to describe the systems used in those studies K6.1 t/m K6.1.3, K62, K6.5 t/K6.5.4.

7.2 Summary of findings from non-clinical studies

Not applicable

7.3 Summary of findings from clinical studies

Not applicable

7.4 Summary of known and potential risks and benefits

The equipment that is to be used in this study is certified by drs. F. Boorsma, head of the department of medical technology of the UMCG and qualified specialist concerning Sterile Medical Devices (DSMH) and matters relating Scope Cleaning and Disinfection (DSRD) (see attachment K6.8.1 and 6.8.2). A FMEA risk analysis has been performed for the SurgVision F2 multispectral imaging system and MDSFR/SFF probe (see attachments K6.2 (SurgVision F2 multispectral imaging system) and K6.5 and K.6.6 (MDSFR/SFF probes)).

7.5 Description and justification of route of administration and dosage

Not applicable

7.6 Dosages, dosage modifications and method of administration

Not applicable

7.7 Preparation and labelling of Non Investigational Medicinal Product

Not applicable

7.8 Drug accountability

Not applicable

8. METHODS

8.1 Study parameters/endpoints

8.1.1 Main study parameter/endpoint

Parameters:

- Macroscopic fluorescent signal levels (tumor to background ratio) and tracer distribution observed by NIR fluorescence imaging
- Macroscopic and real-time quantification of the fluorescent signal observed by means of the MDSFR/SFF spectroscopy probe (M/m^3)
- Biopsy specimen characteristics (number of biopsies, number of positive wound bed biopsies

Reference standards

- Histologically ascertained tissue types (qualitative):
- Normal oral cavity tissue
- Squamous cell carcinoma
- Tissue EGFR levels (immunohistochemistry) and additional immunohistochemical staining.
- To correlate the fluorescent signal assessed by NIR fluorescence endoscopy with other biological and molecular parameters (IHC) and the fluorescent signal assessed in the ex vivo biopsy specimens.

8.1.2 Other study parameters (if applicable)

Patient characteristics (age, sex, BMI, history and morbidity, localization and classification of cancer, treatment outcome, blood pressure, pulse and temperature before and after tracer administration, baseline blood count/ liver and kidney function, signs and symptoms before and after tracer administration.

Histopathologic examinations related to *ex vivo* EGFR expression and cetuximab-IRDyeCW800 distribution.

8.2 Randomization, blinding and treatment allocation

The current study is a non-randomized, non-blinded single center study, in which all patients undergo the same surgical procedure according to standard care. For part I of this study there will be a difference in administered tracer dose; the first 9 patients will be in the 3

different dose-finding arms (10-25-50mg Cetuximab-IRDye800CW). After the inclusion of these 9 patients, and if the dosage is found to be sufficient, we include the next 70 patients. Here, there's no randomization, blinding or treatment allocation applicable since this is a prospective cross sectional diagnostic study.

8.3 Study procedures

General clinical practice will have priority over study procedures at all times.

.

Potential eligible patients are identified by their treating physician (in Dutch:

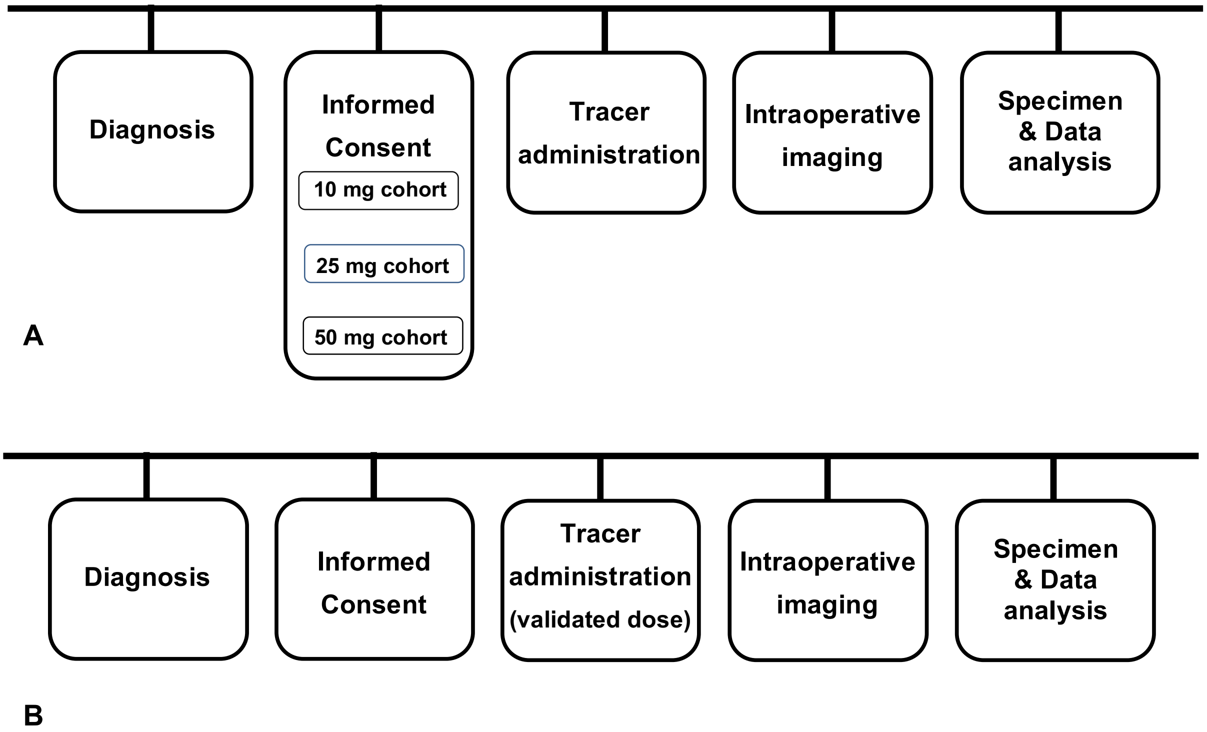

Figure 6 A & B **Study Flow Chart with A representing Part 1 (dose finding) and B representing Part 2.**

hoofdbehandelaar) using the EPD (Electronic patients file (in Dutch: Electronisch Patienten Dossier)) database of the UMCG where all patients with HNSCC are registered. Eligibility is assessed by the research physician (in Dutch: onderzoeksarts) in consultation with the treating physician by checking in- and exclusion criteria based on the available data (according to paragraph 4.2 and 4.3). If the patient is eligible to participate in the study, the treating physician discusses the option to participate in the study. After this, patients will receive a letter containing information about the study and study procedures during the consult or they will receive it by mail when the letter is sent to the patient together with the

standard information each patient receives prior before the scheduled operation (see patient information leaflets for UMC Groningen).

The patients will be informed about the aims of the study, the possible adverse events, the procedures and possible hazards to which they will be exposed before enrolment into the study. They will be informed as to the strict confidentiality of their patient data.

Patients will be contacted by phone, mail or e-mail to ask if they are interested in participating in this study by one of the investigators (treating/research physician) involved in this study.

A flow chart from part 1 and part 2 of the study is provided in Figure 6. For more specified information about informed consent, see chapter 11.2.

Visit 1:

Tracer administration and safety monitoring will occur at the day of administration of the tracer, the patient is asked for signs/symptoms present.

Before administration of the tracer, a pretreatment ECG is performed and vital signs will be measured. A QT prolongation of >440 ms in males or >450 ms in females is not to be accepted and patients will be excluded from further study participation. An intravenous line will be installed. Blood will be drawn during the pre-operative screening, will be used as baseline parameters in case of any adverse events after administration. A blood sample will be taken prior to infusion of cetuximab-IRDye800CW to determine baseline plasma concentration (T=0) (only in dose-escalating cohort). A single dose of 10-25-50 (depending on cohort) will be administered intravenously to the patient. The infusion line will be flushed with a saline solution afterwards. After tracer administration, blood pressure, pulse and temperature will be measured. A second blood sample will be taken to determine plasma concentration and tracer stability one hour after administration (T=1) (only in dose-escalating cohort). All above will be registered by filling in the CRF. The patient will be observed for 60 minutes following tracer injection. During safety monitoring, a crash car with necessary equipment is available in case of an adverse reaction. All above procedures will be performed by the research physician.

Visit 2

One day prior to surgery

The patient will be hospitalized, as standard surgery protocol requires, and is asked for signs/symptoms that occurred since the tracer administration or the last visit. Vital signs will

be measured. Fluorescence images and MDSRF spectra will be acquired of tumor and normal tissue around the tumor region (only in dose-escalating cohort), above will be performed by research physician. A third blood sample will be taken to determine plasma concentration and tracer stability (T=2) (only in dose-escalating cohort).

Day of surgery

The patient is asked for signs/symptoms that occurred since the tracer administration or the last visit. The patient will be brought to narcosis following the standard protocol within the UMCG. A fourth blood sample will be taken to determine plasma concentration and tracer stability (T=3) (only in dose-escalating cohort). A technical assistant will be present to operate the SurgVision multispectral camera and to assure relevant images are saved in close communication with the surgeon (treating physician). Fluorescence images and MDSRF spectra will be acquired of tumor and normal tissue around the tumor region, and the tumor will be excised following the standard protocol. After tumor removal, the woundbed will be inspected and again fluorescence images and MDSRF spectra will be applied. Fluorescent areas in the woundbed will be analyzed by MDSFR spectroscopy and regions of interest will be biopsied. There will be no re-resection as a result of the new obtained biopsy data. Subsequently these biopsies will be processed separately for histopathology and fluorescence microscopy.. Decisions on post-operative strategies will otherwise not be influenced by this study.

After the operation patient will go to the nursing ward and will be strictly observed following the UMCG post-operative protocol. On the first and second day post-operative, blood samples will be drawn to determine plasma concentration and tracer stability (T=4 and T=5) (only in dose-escalating cohort).

Specimen related study protocol

After completion of the resection, the resected specimen will be analyzed immediately after removal by using ex vivo fluorescence imaging and MDSFR spectroscopy. On the excised tumor specimen we will acquire ex vivo fluorescence images and perform MDSFR-point spectroscopy of carefully selected regions of fluorescence. The regions that display cetuximab-IRDye800CW-fluorescence will be inked with non-fluorescent ink after ex vivo fluorescence imaging. These marked areas will be identified by the pathologist for histopathology analysis for the presence of tumor in the subsequent fluorescence microscopy. Important to state, the specimen processing of the excised tumor will be done with no interference with standard of care.

The excised tumor specimen and the separately taken biopsies from the surgical woundbed will be submitted for standard HE histopathology, for assessing the margin status and other descriptive data necessary for clinical use. In the unexpected case that the study-based separately sampled biopsies from the surgical woundbed show tumor, this finding will be used for the decision by the Head and Neck tumor board on post-operative strategy. Decisions on post-operative strategies will otherwise not be influenced by this study. Next to assessment of the margin status by routine histopathology, the inked areas will be specifically analyzed for margin status. Diagnosis of the separately taken biopsies from the woundbed will be done by routine histopathology. After completion of the routine histopathology, the fluorescence microscopy will be performed on all excised tumors and biopsies. Regions of interest (ROI) will be determined on the histological slides and the fluorescence images. The ROI will be assessed by software using algorithms to quantitate the signals. This will separate background signals from tumor related cetuximab-IRDye800CW fluorescence. The complete workflow of the specimen after excision can be summarized:

1. Marking of the fluorescent areas in the OR by ink after ex vivo analysis of the excised tumor specimen.
2. Fixation of tissue in formalin (this will not interfere with fluorescence microscopy, see preliminary data).
3. Laminating of specimen in evenly sized sections of 4 mm.
4. Paraffin embedding of the 4 mm sections.

6) Documenting all slides by regular photography.
7) Tissue sectioning for routine histopathological diagnosis
8) NIR-fluorescence imaging of the paraffin embedded tissue blocks
9) Sectioning and glass mounting of the paraffin blocks in 4 μm thick sections
10) Deparaffinazation in xylene of the 4 μm sections
11) Whole slide fluorescence scanning of all the slices with the Odyssey (21 μm resolution)
12) High resolution fluorescence microscopy of selected tumor and non-tumor areas with a Leica NIR fluorescence microscope
13) Staining of the scanned slides with HE and immunohistochemical staining for EGFR and relevant tumor markers for SCC.
14) Digital whole slide scanning of HE and immunohistochemical stained slides.
15) Overlay and matching of fluorescence image (cetuximab-IRDye800CW) and digital whole slide image of histology and immunohistochemistry for correlation of the tumor and non-tumor areas vs the cetuximab-IRDye800CW fluorescence signal.

16) Spatial reconstruction of the distribution of the cetuximab-IRDye800CW fluorescence.

8.4 Withdrawal of individual subjects

Subjects can leave the study at any time for any reason if they wish to do so without any consequences. The investigator can decide to withdraw a subject due to failure of tracer administration or urgent medical reasons. Direct withdrawal should be considered in case of a serious adverse event.

8.4.1 Specific criteria for withdrawal (if applicable)

Not applicable.

8.5 Replacement of individual subjects after withdrawal

Patient who are withdrawn will be replaced in this study.

8.6 Follow-up of subjects withdrawn from treatment

After administration of the tracer, (serious) adverse events that occur in subjects that are withdrawn from the study procedure, will still be recorded if possible.

8.7 Premature termination of the study

8.7.1 Termination based on safety aspects

A multidisciplinary team with study investigators will discuss safety aspects during the study procedures. Results will be reported immediately in case of any SUSAR to the external Data Safety Monitoring Board (DSMB). After the first 9 patients of part 1 and whenever (serious) adverse events ((S)AE) happen, these will be reported to the DSMB. The study will be considered to be terminated in case a suspected unexpected serious adverse reaction (SUSAR) occurs in any of the patients, according to advice of the DSMB.

8.7.2 Termination based on Cetuximab-IRDye800CW accumulation

This study will be suspended immediately if any serious adverse event related to the administration of the tracer occurs in any of the patients. The design of this study warrants maximal data collection, while risks and burden for patients are minimized. Also, the framework of this study can be used for evaluation of other, newly developed (fluorescent) tracers.

The study will not be terminated if after interim analysis of the first 9 evaluable patients no uptake of Cetuximab-IRDye800CW in SCC tissue can be shown by any of the available technologies; if this occurs the study will continue with inclusion of patients in a new cohort of dose finding

8.7.3 Termination based on other aspects

The study will be suspended based on urgent medical or ethical considerations as decided by the principal investigators. In case of termination of the study, the institution, regulatory authorities, CCMO and the METC of the study center will be informed.

9. SAFETY REPORTING

9.1 Temporary halt for reasons of subject safety

In accordance to section 10, subsection 4, of the WMO, the sponsor will suspend the study if there is sufficient ground that continuation of the study will jeopardize subject health or safety. The sponsor will notify the accredited METC without undue delay of a temporary halt including the reason for such an action. The study will be suspended pending a further positive decision by the accredited METC. The investigator will take care that all subjects are kept informed.

9.2 AEs, SAEs and SUSARs

9.2.1 Adverse events (AEs)

Adverse events are defined as any undesirable experience occurring to a subject during the study, whether or not considered related to [the investigational product / trial procedure/ the experimental intervention]. All adverse events reported spontaneously by the subject or observed by the investigator or his staff will be recorded.

9.2.2 Serious adverse events (SAEs)

A serious adverse event is any untoward medical occurrence or effect that

- results in death;
- is life threatening (at the time of the event);
- requires hospitalization or prolongation of existing inpatients' hospitalization;
- results in persistent or significant disability or incapacity;
- is a congenital anomaly or birth defect; or

- any other important medical event that did not result in any of the outcomes listed above due to medical or surgical intervention but could have been based upon appropriate judgement by the investigator.

An elective hospital admission will not be considered as a serious adverse event.

The sponsor will report the SAEs through the web portal *ToetsingOnline* to the accredited METC that approved the protocol, within 7 days of first knowledge for SAEs that result in death or are life threatening followed by a period of maximum of 8 days to complete the initial preliminary report. All other SAEs will be reported within a period of maximum 15 days after the sponsor has first knowledge of the serious adverse events.

9.2.3 Suspected unexpected serious adverse reactions (SUSARs)

Adverse reactions are all untoward and unintended responses to an investigational product related to any dose administered. Unexpected adverse reactions are SUSARs if the following three conditions are met:

1. The event must be serious (see chapter 9.2.2);
2. There must be a certain degree of probability that the event is a harmful and an undesirable reaction to the medicinal product under investigation, regardless of the administered dose;
3. the adverse reaction must be unexpected, that is to say, the nature and severity of the adverse reaction are not in agreement with the product information as recorded in:

- Summary of Product Characteristics (SPC) for an authorized medicinal product;
- Investigator's Brochure for an unauthorized medicinal product.

The sponsor will report expedited the following SUSARs through the web portal *ToetsingOnline* to the METC:

– SUSARs that have arisen in the clinical trial that was assessed by the METC;
– SUSARs that have arisen in other clinical trials of the same sponsor and with the same medicinal product, and that could have consequences for the safety of the subjects involved in the clinical trial that was assessed by the METC.

The remaining SUSARs are recorded in an overview list (line-listing) that will be submitted once every half year to the METC. This line-listing provides an overview of all SUSARs from the study medicine, accompanied by a brief report highlighting the main points of concern.

The expedited reporting of SUSARs through the web portal Eudravigilance or ToetsingOnline is sufficient as notification to the competent authority.

The sponsor will report expedited all SUSARs to the competent authorities in other Member States, according to the requirements of the Member States.

The expedited reporting will occur not later than 15 days after the sponsor has first knowledge of the adverse reactions. For fatal or life threatening cases the term will be maximal 7 days for a preliminary report with another 8 days for completion of the report.

9.3 Annual safety report

In addition to the expedited reporting of SUSARs, the sponsor will submit, once a year throughout the clinical trial, a safety report to the accredited METC, competent authority, and competent authorities of the concerned Member States.
This safety report consists of:

- a list of all suspected (unexpected or expected) serious adverse reactions, along with an aggregated summary table of all reported serious adverse reactions, ordered by organ system, per study;
- a report concerning the safety of the subjects, consisting of a complete safety analysis and an evaluation of the balance between the efficacy and the harmfulness of the medicine under investigation.

9.4 Follow-up of adverse events

All AEs will be followed until they have abated, or until a stable situation has been reached. Depending on the event, follow up may require additional tests or medical procedures as indicated, and/or referral to the general physician or a medical specialist.
SAEs need to be reported till end of study within the Netherlands, as defined in the protocol

9.5 Data Safety Monitoring Board (DSMB)

An independent external Data Safety Monitoring Board of experts is established to perform ongoing safety surveillance and to evaluate interim analyses on the safety data. This board is necessary considering the available knowledge about Cetuximab-IRDye800CW, see chapter 13.
The independent members of the DSMB are:

- Prof. dr. A.K.L. Reyners, Medical Oncologist, UMCG
- Prof. dr. H.W. Nijman, Gynecologist-oncologist, UMCG
- Dr. M. Nijsten, Intensivist, UMCG

The members of the DSMB have expertise from different scientific areas and are experienced in serving on a DSMB. They have no conflicts of interest with the conducted trial or principal investigators of the study. They have no financial interest in the outcome nor will be authors of future publications of this study. However, we are aware of the fact that the DSMB members work for the same sponsor and in some cases at the same department as the investigators.
The investigators involved in this study will perform an interim analysis on safety data, after the first cohort of 9 patients. Interim analyses are performed according to chapter 10.4. The investigators will report to the independent DSMB. The DSMB will consider essential parts of study conducts like protocol adherence, patient withdrawal and safety. The DSMB will work according to Standard Operating Procedures (SOPs). A DSMB charter is available.

The responsibilities of the DSMB include:

- Monitor protocol compliance by participants and investigators and monitor patient withdrawal as early indicators for problems with respect to safety or feasibility;
- Monitor safety of the tracer Cetuximab-IRDye800CW (e.g. toxicity data, SAEs, deaths);
- Advice on the need for dose adjustments because of safety issues;
- Advise on protocol modifications suggested by investigators or sponsors (e.g. to inclusion criteria, trial endpoints, or sample size);
- Monitor compliance with previous DSMB recommendations;
- Considering the ethical implications of any recommendations made by the DSMB;
- Provide recommendations regarding study modification, continuation or termination, based on the results of the interim analysis;
- Decide whether to recommend that the trial continues to recruit participants or whether recruitment should be terminated either for everyone or for some participant subgroups.

The DSMB will discuss the results of the interim-analysis and advice the steering committee. The DSMB provides recommendations regarding study modification, continuation or termination. Discontinuation of the trial is advised by the DSMB according to the pre-defined stopping guidelines stated in paragraph 8.7. The advice(s) of the DSMB will only be sent to the sponsor of the study. Should the sponsor decide not to fully implement the advice of the DSMB, the sponsor will send the advice to the reviewing METC, including a note to substantiate why (part of) the advice of the DSMB will not be followed.

10. STATISTICAL ANALYSIS

10.1 Descriptive statistics

Patient descriptive data will include TNM status, tumour size, localization, margin status. Only during the dose finding study, integrity of cetuximab-IRDye800CW will be monitored and described.

10.2 Study parameters

The primary aim of part 1 is to determine whether the average **TBR** as observed in 9 patients is sufficient. The primary aim of part 2 of the study is to define a threshold value for fluorescence imaging-guided spectroscopy signal that allows the detection of tumour involved deep margins with high sensitivity while ensuring an adequate positive predictive value. For this we will first relate – on a region of interest (ROI)-based level – the quantitative spectroscopy results (index test) with histopathology (reference standard) to estimate the fraction true and false positives (i.e. spectroscopy positive and histopathology positive respectively negative), and fraction false negatives (i.e. spectroscopy negative and histopathology positive). If a tumour-positive margin was not evaluated by fluorescence-imaging guided spectroscopy it will be considered spectroscopy negative. The fraction true negatives cannot be taken into account here, as the number of true negative ROIs in each patient is unidentifiable. From these data we can calculate the sensitivity and positive predictive value at all possible threshold values of the spectroscopy signal, leading to a precision-recall curve from which we will derive the optimal threshold value. As each patient may contribute multiple ROIs – thereby increasing statistical power –, we will take clustering into account by multilevel analysis. Furthermore, we will include bootstrapping methods for the threshold finding to correct for optimism (i.e. to ensure that the proposed threshold will not only hold true in the data it derived from, but also in new patients).

Next, we will evaluate the defined spectroscopy threshold value on a per-patient level (true positive: positive margins with all corresponding ROIs spectroscopy positive, regardless of false-positive ROIs; false negative: positive margins with one or more corresponding ROIs spectroscopy negative, regardless of false-positive ROIs; true negative: negative margins and no ROI spectroscopy positive; false positives: all others). From these data we will calculate the sensitivity, specificity, positive and negative predictive value, together with corresponding 95% confidence intervals. This analysis will inform to what extent the number

of positive margins as occurred during regular surgery could possibly have been lower if a re-resection immediately could have been considered guided by the intraoperative imaging results.

10.3 Interim analysis

An interim analysis towards the primary endpoint will be conducted by the investigators involved in this study after the first 9 patients (3 patients per dosage) of the dose finding study. If no adequate tumor-to-background ratios are observed in the first 9 patients with HNSCC, the dose finding study might be extended.

An interim analysis towards the key safety parameters including cetuximab-IRDye800CW induced AE's, SAE's and SUSARS will be performed after the first 9 patients (see also the DSMB charter and sections 8.7.2 and 9.5 of this protocol). The results will be reviewed by the external independent data monitoring committee (DSMB).

11. ETHICAL CONSIDERATIONS

11.1 Regulation statement

The study will be conducted according to the principles of the Declaration of Helsinki (Fortaleza, Brasil, 2013 amendment) and in accordance with the medical Research Involving Human Subjects Act (WMO) and other guidelines, regulations and Acts. The protocol has been written and the study will be conducted according to the ICH Harmonized Tripartite Guideline for Good Clinical Practice. The protocol will be approved by the Local, Regional or National Ethics Committees.

11.2 Recruitment and consent

Potential eligible patients are identified by their treating physician (in Dutch: hoofdbehandelaar) using the EPD database of the UMCG where all patients with HNSCC are registered. Eligibility is assessed by the research physician (in Dutch: onderzoeksarts) in consultation with the treating physician by checking in- and exclusion criteria based on the available data (according to paragraph 4.2 and 4.3). If the patient is eligible to participate in the study, the treating physician discusses the option to participate in the study. After this, patients will receive a letter containing information about the study and study procedures during the consult or they will receive it by mail when the letter is sent to the patient together

with the standard information each patient receives prior before the scheduled operation (see patient information leaflets for UMC Groningen).

.

The patients will be informed about the aims of the study, the possible adverse events, the procedures and possible hazards to which they will be exposed before enrolment into the study. They will be informed as to the strict confidentiality of their patient data.
Patients will be contacted by phone, mail or e-mail to ask if they are interested in participating in this study by one of the investigators (treating/research physician) involved in this study.

Each patient will be given the opportunity to ask questions and will be informed about the right to withdraw from the study at any time without prejudice. See the patient information sheet and patient informed consent statement for the UMC Groningen.
When patients intend to participate, the visit for tracer administration with a pretreatment ECG is planned and patients will be asked to bring the signed informed consent form, which will be received by the research physician.

Informed consent

Documented informed consent must be obtained for all patients included in the study before they are registered in the study. Patients must be given adequate opportunity to read the information and enquire about details of the study before consent is given. The informed consent procedure takes place conform the ICH guidelines on Good Clinical Practice. This implies that the written informed consent form will be signed and personally dated by the patient or by the patient's legally acceptable representative. The informed consent statement will be signed and dated by the research physician afterwards and the patient will receive a copy. The general physician of each patient will be informed about the enrolment of the patient to the study.

11.3 Objection by minors or incapacitated subjects (if applicable)

Not applicable

11.4 Benefits and risks assessment, group relatedness

For the participating patients, there is no diagnostic or treatment benefit related to the study. Participation may possibly produce useful scientific data for the future. Risks related to the

administration of cetuximab-IRDye800CW are described in the IMPD (version 1.0, July 2016, section 2.4). The risks related to cetuximab-IRDye800 CW administration are extensively described in section 6.1. The risks of the surgical treatment are comparable to a clinical surgical treatment for HNSCC (without the study protocol) the risk of the NIR fluorescence imaging procedure and spectroscopy procedure is very minimal, as shown in the preliminary data (UAB). The surgical planning will not be altered due to the fluorescence imaging during this study. During surgery, delineation of margins will not be influenced by the intraoperative imaging data. The fluorescence imaging will not influence surgical decisions. The type of surgery will not be influenced by the study protocol.

11.5 Compensation for injury

See attachment G1 and G2
Briefly, the sponsor/investigator has a liability insurance which is in accordance with article 7 of the WMO.
The sponsor (also) has an insurance which is in accordance with the legal requirements in the Netherlands (Article 7 WMO). This insurance provides cover for damage to research subjects through injury or death caused by the study.

1. € 650,000.-- (i.e. six hundred and fifty thousand Euro) for death or injury for each subject who participates in the Research;
2. € 5,000,000.-- (i.e. five million five hundred thousand Euro) for death or injury for all subjects who participate in the Research;
3. € 7,500,000.-- (i.e. seven million five hundred thousand Euro) for the total damage incurred by the organization for all damage disclosed by scientific research for the Sponsor as 'verrichter' in the meaning of said. Act in each year of insurance coverage.

The insurance applies to the damage that becomes apparent during the study or within 4 years after the end of the study.

11.6 Incentives (if applicable)

For each day of patient related study procedures, the subjects will receive compensation for travelling expenses (€ 0.19/km) and a ticket for free parking.

12. ADMINISTRATIVE ASPECTS, MONITORING AND PUBLICATION

12.1 Handling and storage of data and documents

Case Record Forms

Case Record Forms (CRFs) will be provided by investigator. The CRFs will be completed by the investigators. The investigators are responsible for the legibility, completeness and correctness of the CRF. The signature of the investigator at the end of each chapter of the CRF will serve as a confirmation. Errors, changes and/or additions entered on original CRFs must be corrected by drawing in a single line through the incorrect entry and writing the new entry as close to the original as possible as so to leave the correct entry legible. If necessary the reason for the change must be given. The correction must be initialed and dated by the authorized person making the change.

Data storage

Data of patients will be handled confidentially and a coded identification number (study protocol number 'ICON' followed by number of inclusion: 01) will be used to link the data to the specific patients. The data that can be linked to a specific patient will be stored separately. The medical investigator safeguards the key to the code. The handling of the personal data complies with the Dutch Personal Data Protection Act (in Dutch: de Wet bescherming persoonsgegevens). These data will be stored at the specific site for at least twenty years. Coded / anonymized study data will be made available to our relevant partners within the project; Erasmus Medical Center, Rotterdam and University Medical Center Utrecht. They will assist in the use and maintenance of the MDSRF spectroscope. Moreover, the will perform parts of the data analysis of the MDSRF spectroscope.

12.2 Monitoring and Quality Assurance

On-site monitoring will take place conform the NFU (Nederlandse Federatie van Universitair Medische Centra)-guideline "Kwaliteitsborging van mensgebonden onderzoek 2010" by the appointed monitor. For this study, the risk classification is considered "high", which implies intensive independent monitoring of at least 3 visits per year, dependent on the patient inclusion speed. This study will be monitored by an independent, certified monitor, employed by the UMC Groningen The monitor will perform source data verification on the research data by comparing the data entered into the CRF with the available source documentation and other available documents. Source documents are defined as the patient's hospital medical records, clinician notes, laboratory print outs, digital and hard copies of imaging, memos, electronic data etc.

Data Verification

The monitor will verify the following items (100%): Patient flow (inclusion speed and dropout rate); Informed consent forms (presence, dates, signatures); Trial Master File and

Investigator Files (presence of all documents); in-/exclusion criteria (using source documents); primary endpoints (safety and validity); SAEs / SUSARs (number, missed, reporting procedures); study product (administration, accountability). Study procedures will be verified by reviewing Standard Operating Procedures and other instructions. Also, the presence of certificates and Standard Operating Procedures of used devices, facilities, laboratories, pharmacies and other departments will be assessed

12.3 Amendments

Amendments are changes made to the research after a favorable opinion by the accredited METC has been given. All amendments will be notified to the METC that gave a favorable opinion.

A 'substantial amendment' is defined as an amendment to the terms of the METC application, or to the protocol or any other supporting documentation, that is likely to affect to a significant degree:

- the safety or physical or mental integrity of the subjects of the trial;
- the scientific value of the trial;
- the conduct or management of the trial; or
- the quality or safety of any intervention used in the trial.

All substantial amendments will be notified to the METC and to the competent authority. Non-substantial amendments will not be notified to the accredited METC and the competent authority, but will be recorded and filed by the sponsor.

12.4 Annual progress report

The sponsor/investigator will submit a summary of the progress of the trial to the accredited METC once a year. Information will be provided on the date of inclusion of the first subject, numbers of subjects included and numbers of subjects that have completed the trial, serious adverse events/ serious adverse reactions, other problems, and amendments.

12.5 Temporary halt and (prematurely) end of study report

The sponsor will notify the accredited METC and the competent authority of the end of the study within a period of 90 days. The end of the study is defined as the last patient's last visit. The sponsor will notify the METC immediately of a temporary halt of the study, including the reason of such an action. In case the study is ended prematurely, the sponsor will notify the accredited METC and the competent authority within 15 days, including the reasons for the

premature termination. Within one year after the end of the study, the investigator/sponsor will submit a final study report with the results of the study, including any publications/abstracts of the study, to the accredited METC and the Competent Authority.

12.6 Public disclosure and publication policy

The financial sponsor of the study is the University Medical Center Groningen (Principal Investigators dr. M.J.H. Witjes, prof. Dr. G.M. van Dam).

13. STRUCTURED RISK ANALYSIS

13.1 Potential issues of concern

a. Level of knowledge about mechanism of action

Cetuximab is an epidermal growth factor receptor (EGFR) inhibitor used for the treatment of metastatic colorectal cancer, metastatic non-small cell lung cancer and head and neck cancer. Evidence suggests that the EGFR is involved in the pathogenesis and progression of different carcinoma types and differentially expressed in normal tissue versus HNSCC. Cetuximab is used extensively in the clinic for its anti-neoplastic properties.
IRDye800CW is designed for antibody, protein, or peptide labeling. IRDye800CW has excitation/emission maxima at 774 nm/789 nm, precisely centered in the region known to give optimal signal-to-noise ratio for optical imaging. It is extensively used in preclinical optical imaging experiments for the tracking of probes, since near-infrared fluorophores minimize the optical challenges of detecting photons in tissues.

b. Previous exposure of human beings with the test product(s) and/or products with a similar biological mechanism

IRDye800CW solely has never been administered to humans, except in combination with bevacizumab-800CW and cetuximab-IRDye800CW. In animals, the administration of 5 mg/kg of IRDye800CW intravenously showed a fairly consistent peak plasma concentrations. Toxicity data obtained with IRDye 800CW was published by Marshall et al. (40). Based on hematologic, clinical chemistry, and histopathologic evaluation, single administration of IRDye800CW carboxylate intravenously at dose levels of 1, 5, and 20 mg/kg (intravenous) or 20 mg/kg (intradermal) produced no pathological evidence of toxicity. A dose of 20 mg/kg

was identified as the no observed adverse effect level following IV or ID routes of administration of IRDye800CW.

Animal toxicological studies on cetuximab-IRDye800CW and preclinical tracer evaluation data showed no adverse effects (45).
More recently, a toxicity study on cetuximab-IRDye800CW has been performed in macaques (Macada fascicularis) by the University of Alabama in Birmingham (UAB) (39). The macaques were dosed at 250 mg/m2 (20.83 mg/kg), the full human therapeutic dose level of cetuximab. Cetuximab- 800CW and cetuximab were tolerated well under the conditions of the study, except for an increased QTc in both groups after dosing. The QTc returned to baseline levels at day 15 for the cetuximab control group, but not for the cetuximab-IRDye800CW group. Moreover, well described side-effects of cetuximab are hypomagnesemia, hypocalcemia and hypokalemia which can also cause a prolonged QT interval. For this reason, ECG and potassium, magnesium and calcium monitoring was included in the phase 1 dose-escalation study of cetuximab-IRDye800CW. The phase 1 dose-escalation study with cetuximab-IRDye800CW was performed in human subjects with head and neck squamous cell carcinoma (NCT01987375). In this study, nine patients received 2.5 mg/m2, 25 mg/m2 or 62.5mg/m2 cetuximab-IRDye800CW, three for each dose level. There were no grade-2 or higher toxicities related to the study drug. There were no grade-2 or higher adverse events attributable to cetuximab-IRDye800CW and four possibly related grade-1 adverse reactions occurred in the first cohort, four in the second cohort, and two in the third cohort. This demonstrated that these toxicities were not dose related. The principal investigator of the study, prof. Eben Rosenthal, concluded that these side-effects are most likely cetuximab related and not related to the conjugate. The QTc interval was significantly (P=0.028) higher at 2 hours compared to pre-infusion values, but returned to baseline at 30-day post-infusion

c. Can the primary or secondary mechanism be induced in animals and/or in *ex-vivo* human cell material?

See section above, 13.1b, for results in recent animal study.

d. Selectivity of the mechanism to target tissue in animals and/or human beings

See IMPD version 1.0, July 2016, section 2.2.1.1.

e. Analysis of potential effect

The safety data of cetuximab are all collected for the therapeutic dose of cetuximab. The

overall safety profile of cetuximab is based on data of over 5,500 with various malignancies, including a significantly percentage with HNSCC, predominantly treated with cetuximab 250-450 mg/m^2 body weight on a weekly interval.

Details on the nature and seriousness of potential adverse effects are described in 'summary of product characteristics' of cetuximab (see attachment D1, section 4.8, page 7-9).

The initial dose for patients with metastatic carcinoma of the head and neck in combination with radiation or chemotherapy is 450 mg/m^2 body, followed by 250 mg/m^2 once a week. However, we start our dose finding study at a level of only 10 mg, as we aim to attain a dose as low as possible, which is still sufficient for imaging purposes and safe for the participants. Not that 10 mg is the absolute dose, and not per kilogram bodyweight.

Potential side effects at a dose on 10-50 mg cetuximab can be summarized as skin rash, hypersensitivity reactions and hypomagnesemia. In the study performed at UAB only grade 1 toxicities occurred.(46)

f. Pharmacokinetic considerations

Binding to IRDye800cw does not alter the binding capacity of cetuximab, pharmacokinetics or bio distribution, as previously shown. (45)

g. Study population

The study population are patients with a primary or recurrent HNSCC who will be treated by surgery. For exact in- and exclusion criteria, see section 4.2 and 4.3 of this protocol.

h. Interaction with other products

Not applicable

i. Predictability of effect

A recent study showed good localization of ^{89}Zirconium labeled cetuximab in HNSCC in PET imaging (38). Based on the preliminary results of our collaborators (UAB), we expect the tracer cetuximab-IRDye800CW to accumulate in EGFR expressing tumors. We will receive direct feedback the moment we visualize the fluorescence in the NIR spectrum using the intra-operative camera. The main advantage of using IRDye800CW for in vivo optical imaging, is the fact that this dye is fluorescent in the near infrared (NIR) spectrum, a spectrum with minimal autofluorescence. Most endogenous fluorophores, in particular hemoglobin, strongly absorb light in the visible light spectrum. Other biological components such as water and lipids strongly absorb light in the infrared region. The combined absorption

wavelength of these components translates into a window from approximately 650 to 900nm, near to the infrared light, where the absorption coefficient, and thus autofluorescence, of tissue is at a minimum. Due to this low autofluorescence, the near infrared spectrum (700-900nm) is the optimal window for in vivo, perioperative, HNSCC imaging.

j. Can effects be managed?

A pretreatment ECG will be performed to assure no QTc prolongation is present. Right before cetuximab-IRDye800CW is administered intravenously, blood pressure, pulse and temperature will be measured. There is extensive experience with the administration of cetuximab and the risk of adverse reactions is very small, therefore we decided to observe the patients in this study 60 minutes following tracer injection. Vital signs will be measured after 15, 30, 45 and 60 minutes. During safety monitoring, a crash car with necessary equipment is available in case of an adverse reaction. After the observational period of 1 hour, blood will be drawn, afterwards the intravenous line will be removed and the patient will be discharged with adequate advice to contact the Oral and Maxillofacial surgeon on call if necessary.

13.2 Synthesis

Animal toxicological studies on cetuximab-IRDye800CW and preclinical tracer evaluation data showed no adverse effects.(45) A recent clinical study with 89zr-cetuximab showed that only known adverse events to cetuximab were observed, such as skin toxicity, hypomagnesaemia and infusion related reactions, none exceeding grade 2.(47) Cetuximab-IRDye800CW has administered intravenously to 9 patients in the earlier mentioned dose-finding study, no toxicity of the tracer bevacizumab-800IRDyeCW was observed in any of the patients.(39) Up until now, no SAE’s or AE’s have been reported. Adverse Events may be expected after administration, based on our experience with administrating a much higher dose of unlabeled cetuximab. Hypersensitivity reactions to cetuximab can occur within a short term after administration (up until 1 hour).

Also, hypo- or hypertension can occur after cetuximab administration. However, the expected adverse events are temporal without clinical consequences. This is why the patients will be monitored for one hour after tracer injection, with measurements of vital signs on regular base.

The study protocol with not interfere with general clinical practice. Therefore, surgical risks of patients included in the study protocol will be comparable to patients undergoing a surgical procedure for HNSCC. If necessary, biopsies will be taken after tumor removal. This has in

general a minimal risk of extra bleeding, however, this is not significant to the already made woundbed after excision of the tumor. Though, if this complication occurs, which is expected to be very uncommon, the surgeon has several tools to handle this problem adequately. Postoperatively, patients will go to the nursing ward and will be strictly observed following the UMCG post-operative protocol. Naturally, the procedure will only take place if this is considered medically justified by the Oral and Maxillofacial surgeon.

14. REFERENCES

(1) Dutch Head & Neck Society guidelines: http://www.oncoline.nl/mondholte-en-orofarynxcarcinoom.

(2) Denaro N, Russi EG, Adamo V, Merlano MC. State-of-the-art and emerging treatment options in the management of head and neck cancer: news from 2013. Oncology 2014;86(4):212-229.

(3) Jonkman A, Kaanders JH, Terhaard CH, Hoebers FJ, van den Ende PL, Wijers OB, et al. Multicenter validation of recursive partitioning analysis classification for patients with squamous cell head and neck carcinoma treated with surgery and postoperative radiotherapy. Int J Radiat Oncol Biol Phys 2007 May 1;68(1):119-125.

(4) Langendijk JA, Ferlito A, Takes RP, Rodrigo JP, Suarez C, Strojan P, et al. Postoperative strategies after primary surgery for squamous cell carcinoma of the head and neck. Oral Oncol 2010 Aug;46(8):577-585.

(5) Woolgar JA, Hall GL. Determinants of outcome following surgery for oral squamous cell carcinoma. Future Oncol 2009 Feb;5(1):51-61.

(6) Hinni ML, Ferlito A, Brandwein-Gensler MS, Takes RP, Silver CE, Westra WH, et al. Surgical margins in head and neck cancer: a contemporary review. Head Neck 2013 Sep;35(9):1362-1370.

(7) Woolgar JA, Triantafyllou A. A histopathological appraisal of surgical margins in oral and oropharyngeal cancer resection specimens. Oral Oncol 2005 Nov;41(10):1034-1043.

(8) Sutton DN, Brown JS, Rogers SN, Vaughan ED, Woolgar JA. The prognostic implications of the surgical margin in oral squamous cell carcinoma. Int J Oral Maxillofac Surg 2003 Feb;32(1):30-34.

(9) Bernier J, Domenge C, Ozsahin M, Matuszewska K, Lefebvre JL, Greiner RH, et al. Postoperative irradiation with or without concomitant chemotherapy for locally advanced head and neck cancer. N Engl J Med 2004 May 6;350(19):1945-1952.

(10) Frowen J, Drosdowsky A, Perry A, Corry J. Long-term swallowing after chemoradiotherapy: Prospective study of functional and patient-reported changes over time. Head Neck 2016 Apr;38 Suppl 1:E307-15.

(11) Ronis DL, Duffy SA, Fowler KE, Khan MJ, Terrell JE. Changes in quality of life over 1 year in patients with head and neck cancer. Arch Otolaryngol Head Neck Surg 2008 Mar;134(3):241-248.

(12) Patel RS, Goldstein DP, Guillemaud J, Bruch GA, Brown D, Gilbert RW, et al. Impact of positive frozen section microscopic tumor cut-through revised to negative on oral carcinoma control and survival rates. Head Neck 2010 Nov;32(11):1444-1451.

(13) Kodama M, Khanal A, Habu M, Iwanaga K, Yoshioka I, Tanaka T, et al. Ultrasonography for intraoperative determination of tumor thickness and resection margin in tongue carcinomas. J Oral Maxillofac Surg 2010 Aug;68(8):1746-1752.

(14) Shaw RJ, Hall GL, Woolgar JA, Lowe D, Rogers SN, Field JK, et al. Quantitative methylation analysis of resection margins and lymph nodes in oral squamous cell carcinoma. Br J Oral Maxillofac Surg 2007 Dec;45(8):617-622.

(15) van Dam GM, Themelis G, Crane LM, Harlaar NJ, Pleijhuis RG, Kelder W, et al. Intraoperative tumor-specific fluorescence imaging in ovarian cancer by folate receptor-alpha targeting: first in-human results. Nat Med 2011 Sep 18;17(10):1315-1319.

(16) Ogawa M, Kosaka N, Choyke PL, Kobayashi H. In vivo molecular imaging of cancer with a quenching near-infrared fluorescent probe using conjugates of monoclonal antibodies and indocyanine green. Cancer Res 2009 Feb 15;69(4):1268-1272.

(17) van der Vorst JR, Schaafsma BE, Verbeek FP, Keereweer S, Jansen JC, van der Velden LA, et al. Near-infrared fluorescence sentinel lymph node mapping of the oral cavity in head and neck cancer patients. Oral Oncol 2013 Jan;49(1):15-19.

(18) Crane LM, Themelis G, Pleijhuis RG, Harlaar NJ, Sarantopoulos A, Arts HJ, et al. Intraoperative multispectral fluorescence imaging for the detection of the sentinel lymph node in cervical cancer: a novel concept. Mol Imaging Biol 2011 Oct;13(5):1043-1049.

(19) Warram JM, de Boer E, Sorace AG, Chung TK, Kim H, Pleijhuis RG, et al. Antibody-based imaging strategies for cancer. Cancer Metastasis Rev 2014 Sep;33(2-3):809-822.

(20) Themelis G, Yoo JS, Soh KS, Schulz R, Ntziachristos V. Real-time intraoperative fluorescence imaging system using light-absorption correction. J Biomed Opt 2009 Nov-Dec;14(6):064012.

(21) Ntziachristos V, Bremer C, Weissleder R. Fluorescence imaging with near-infrared light: new technological advances that enable in vivo molecular imaging. Eur Radiol 2003 Jan;13(1):195-208.

(22) Ntziachristos V, Ripoll J, Wang LV, Weissleder R. Looking and listening to light: the evolution of whole-body photonic imaging. Nat Biotechnol 2005 Mar;23(3):313-320.

(23) Keereweer S, Van Driel PB, Snoeks TJ, Kerrebijn JD, Baatenburg de Jong RJ, Vahrmeijer AL, et al. Optical image-guided cancer surgery: challenges and limitations. Clin Cancer Res 2013 Jul 15;19(14):3745-3754.

(24) Amelink A, van der Ploeg van den Heuvel,A., de Wolf WJ, Robinson DJ, Sterenborg HJ. Monitoring PDT by means of superficial reflectance spectroscopy. J Photochem Photobiol B 2005 Jun 1;79(3):243-251.

(25) Amelink A, Bard MP, Burgers SA, Sterenborg HJ. Single-scattering spectroscopy for the endoscopic analysis of particle size in superficial layers of turbid media. Appl Opt 2003 Jul 1;42(19):4095-4101.

(26) Kanick SC, Robinson DJ, Sterenborg HJ, Amelink A. Method to quantitate absorption coefficients from single fiber reflectance spectra without knowledge of the scattering properties. Opt Lett 2011 Aug 1;36(15):2791-2793.

(27) Kanick SC, Sterenborg HJ, Amelink A. Empirical model of the photon path length for a single fiber reflectance spectroscopy device. Opt Express 2009 Jan 19;17(2):860-871.

(28) Kanick SC, Gamm UA, Sterenborg HJ, Robinson DJ, Amelink A. Method to quantitatively estimate wavelength-dependent scattering properties from multidiameter single fiber reflectance spectra measured in a turbid medium. Opt Lett 2011 Aug 1;36(15):2997-2999.

(29) Kanick SC, Robinson DJ, Sterenborg HJ, Amelink A. Semi-empirical model of the effect of scattering on single fiber fluorescence intensity measured on a turbid medium. Biomed Opt Express 2012 Jan 1;3(1):137-152.

(30) Kanick SC, Gamm UA, Schouten M, Sterenborg HJ, Robinson DJ, Amelink A. Measurement of the reduced scattering coefficient of turbid media using single fiber reflectance spectroscopy: fiber diameter and phase function dependence. Biomed Opt Express 2011 Jun 1;2(6):1687-1702.

(31) Gamm UA, Kanick SC, Sterenborg HJ, Robinson DJ, Amelink A. Quantification of the reduced scattering coefficient and phase-function-dependent parameter gamma of turbid media using multidiameter single fiber reflectance spectroscopy: experimental validation. Opt Lett 2012 Jun 1;37(11):1838-1840.

(32) Gamm UA, Kanick SC, Sterenborg HJ, Robinson DJ, Amelink A. Measurement of tissue scattering properties using multi-diameter single fiber reflectance spectroscopy: in silico sensitivity analysis. Biomed Opt Express 2011 Nov 1;2(11):3150-3166.

(33) Bauman JE, Ferris RL. Integrating novel therapeutic monoclonal antibodies into the management of head and neck cancer. Cancer 2014 Mar 1;120(5):624-632.

(34) Terwisscha van Scheltinga AG, van Dam GM, Nagengast WB, Ntziachristos V, Hollema H, Herek JL, et al. Intraoperative near-infrared fluorescence tumor imaging with vascular endothelial growth factor and human epidermal growth factor receptor 2 targeting antibodies. J Nucl Med 2011 Nov;52(11):1778-1785.

(35) Grandis JR, Tweardy DJ. TGF-alpha and EGFR in head and neck cancer. J Cell Biochem Suppl 1993;17F:188-191.

(36) Ferrara N, Davis-Smyth T. The biology of vascular endothelial growth factor. Endocr Rev 1997 Feb;18(1):4-25.

(37) Psyrri A, Yu Z, Weinberger PM, Sasaki C, Haffty B, Camp R, et al. Quantitative determination of nuclear and cytoplasmic epidermal growth factor receptor expression in oropharyngeal squamous cell cancer by using automated quantitative analysis. Clin Cancer Res 2005 Aug 15;11(16):5856-5862.

(38) Borjesson PK, Postema EJ, de Bree R, Roos JC, Leemans CR, Kairemo KJ, et al. Radioimmunodetection and radioimmunotherapy of head and neck cancer. Oral Oncol 2004 Sep;40(8):761-772.

(39) de Boer E, Warram JM, Tucker MD, Hartman YE, Moore LS, de Jong JS, et al. In Vivo Fluorescence Immunohistochemistry: Localization of Fluorescently Labeled Cetuximab in Squamous Cell Carcinomas. Sci Rep 2015 Jun 29;5:10169.

(40) Marshall MV, Draney D, Sevick-Muraca EM, Olive DM. Single-dose intravenous toxicity study of IRDye 800CW in Sprague-Dawley rats. Mol Imaging Biol 2010 Dec;12(6):583-594.

(41) Kanick SC, Robinson DJ, Sterenborg HJ, Amelink A. Extraction of intrinsic fluorescence from single fiber fluorescence measurements on a turbid medium. Opt Lett 2012 Mar 1;37(5):948-950.

(42) Kanick SC, Robinson DJ, Sterenborg HJ, Amelink A. Monte Carlo analysis of single fiber reflectance spectroscopy: photon path length and sampling depth. Phys Med Biol 2009 Nov 21;54(22):6991-7008.

(43) Kanick SC, Sterenborg HJ, Amelink A. Empirical model of the photon path length for a single fiber reflectance spectroscopy device. Opt Express 2009 Jan 19;17(2):860-871.

(44) van Leeuwen-van Zaane F, Gamm UA, van Driel PB, Snoeks TJ, de Bruijn HS, van der Ploeg-van den Heuvel,A., et al. Intrinsic photosensitizer fluorescence measured using multi-diameter single-fiber spectroscopy in vivo. J Biomed Opt 2014 Jan;19(1):15010.

(45) Zinn KR, Korb M, Samuel S, Warram JM, Dion D, Killingsworth C, et al. IND-directed safety and biodistribution study of intravenously injected cetuximab-IRDye800 in cynomolgus macaques. Mol Imaging Biol 2015 Feb;17(1):49-57.

(46) Rosenthal EL, Warram JM, de Boer E, Chung TK, Korb ML, Brandwein-Gensler M, et al. Safety and Tumor Specificity of Cetuximab-IRDye800 for Surgical Navigation in Head and Neck Cancer. Clin Cancer Res 2015 Aug 15;21(16):3658-3666.

(47) Menke-van der Houven van Oordt,C.W., Gootjes EC, Huisman MC, Vugts DJ, Roth C, Luik AM, et al. 89Zr-cetuximab PET imaging in patients with advanced colorectal cancer. Oncotarget 2015 Oct 6;6(30):30384-30393.
