# Supplementary material for: EGFR-targeted fluorescence molecular imaging for intraoperative margin assessment in oral cancer patients: a phase II trial
Source: Nat Commun. 2023 Aug 16;14:4952. doi: 10.1038/s41467-023-40324-8 (PMC10432510; doi:10.1038/s41467-023-40324-8)
Supplement: Supplementary file 3 — Reporting Summary [file 41467_2023_40324_MOESM3_ESM.pdf]

nature portfolio

Corresponding author(s): M.J.H. Witjes

Last updated by author(s): Jul 10, 2023

Reporting Summary

Nature Portfolio wishes to improve the reproducibility of the work that we publish. This form provides structure for consistency and transparency in reporting. For further information on Nature Portfolio policies, see our Editorial Policies and the Editorial Policy Checklist.

Statistics

For all statistical analyses, confirm that the following items are present in the figure legend, table legend, main text, or Methods section.

n/a	Confirmed	
☐	☒	The exact sample size (*n*) for each experimental group/condition, given as a discrete number and unit of measurement
☐	☒	A statement on whether measurements were taken from distinct samples or whether the same sample was measured repeatedly
☐	☒	The statistical test(s) used AND whether they are one- or two-sided<br>*Only common tests should be described solely by name; describe more complex techniques in the Methods section.*
☒	☐	A description of all covariates tested
☐	☒	A description of any assumptions or corrections, such as tests of normality and adjustment for multiple comparisons
☐	☒	A full description of the statistical parameters including central tendency (e.g. means) or other basic estimates (e.g. regression coefficient) AND variation (e.g. standard deviation) or associated estimates of uncertainty (e.g. confidence intervals)
☐	☒	For null hypothesis testing, the test statistic (e.g. *F*, *t*, *r*) with confidence intervals, effect sizes, degrees of freedom and *P* value noted<br>*Give P values as exact values whenever suitable.*
☒	☐	For Bayesian analysis, information on the choice of priors and Markov chain Monte Carlo settings
☒	☐	For hierarchical and complex designs, identification of the appropriate level for tests and full reporting of outcomes
☐	☒	Estimates of effect sizes (e.g. Cohen's *d*, Pearson's *r*), indicating how they were calculated

Our web collection on statistics for biologists contains articles on many of the points above.

Software and code

Policy information about availability of computer code

Data collection	Two fluorescent camera systems were used; The Pearl Trilogy (LI-COR Biosciences, Lincoln, NE, USA), which makes use of Image Studio Software Lite version 5.0, and the SurgVision Open Air (SurgVision GmbH, Munich, Germany) which uses dedictared custom software.
Data analysis	For data analysis, GraphPad Prism (version 8.0, GraphPad Software Inc, San Diego, California, USA) and R (version 4.2.2. for MacOS, R Foundation for Statistical Computing, Vienna, Austria) were used for statistical analysis and graph design. ImageJ Fiji (Version 2.3.0/1.53f) was used for fluorescence image analysis. No other open or commercial codes were used.

For manuscripts utilizing custom algorithms or software that are central to the research but not yet described in published literature, software must be made available to editors and reviewers. We strongly encourage code deposition in a community repository (e.g. GitHub). See the Nature Portfolio guidelines for submitting code & software for further information.

Data

Policy information about availability of data

All manuscripts must include a data availability statement. This statement should provide the following information, where applicable:

- Accession codes, unique identifiers, or web links for publicly available datasets
- A description of any restrictions on data availability
- For clinical datasets or third party data, please ensure that the statement adheres to our policy

All imaging data, safety data, clinical details and laboratory data (i.e. restricted to non-identifying data) are available from the corresponding author on request.

Data can be inquired by the corresponding author (M. J. H. Witjes, m.j.h.witjes@umcg.nl). The data will be saved for a minimum of 20 years, in concordance to the Dutch legislations. Upon request, data can be made available to third parties for up to six weeks. The source data for the figures are provided in the source data files. All other data are available within the Article and Supplementary information, including the research protocol. All measurements were taken from distinct samples.

Research involving human participants, their data, or biological material

Policy information about studies with human participants or human data. See also policy information about sex, gender (identity/presentation), and sexual orientation and race, ethnicity and racism.

Reporting on sex and gender	We have used terms sex and gender according to current standard. Source data regarding sex/gender was our elektronical patient file. Our findings apply to both genders. No selection was made based on gender, 50% of patients were female. Patients' permission was obtained to publish (anonymized) personal data.
Reporting on race, ethnicity, or other socially relevant groupings	No preselection was made based on gender or ethnicity, or any other socially relevant groupings.
Population characteristics	Patients' age, sex and history of oral cancer treatment (surgery/radiotherapy) was reported. We included all human subjects regardless of sex/gender or ethnicity. All subjects aged >18 were eligible, no selection was made based on age.
Recruitment	Patients were informed by JdW, JV, FV, MW, KP or SdV. Patients had at least one week to decide to participate after written and oral information was provided. Written informed consent was obtained before any study related procedures. Patients were excluded if they presented with a life expectancy of <12 weeks, Karnofsky performance status <70%, history of infusion reactions to monoclonal antibody therapies, QT prolongation on screening electrocardiogram, uncontrolled medical conditions or episodes within six months prior to enrolment (including uncontrolled hypertension, cerebrovascular accident, significant cardiopulmonary and liver disease), pregnancy, abnormal electrolyte status, use of class IA or III antiarrhythmic drug, or administration of an investigational drug within 30 days prior to the infusion of cetuximab-800CW. Therefore we do not expect any (selection) biases.
Ethics oversight	The study protocol was approved by the medical ethical review committee (METc) at the University Medical Centre Groningen (UMCG) (METc 2016/395).

Note that full information on the approval of the study protocol must also be provided in the manuscript.

Field-specific reporting

Please select the one below that is the best fit for your research. If you are not sure, read the appropriate sections before making your selection.

☒ Life sciences ☐ Behavioural & social sciences ☐ Ecological, evolutionary & environmental sciences

For a reference copy of the document with all sections, see nature.com/documents/nr-reporting-summary-flat.pdf

Life sciences study design

All studies must disclose on these points even when the disclosure is negative.

Sample size	Historical data in our centre showed a tumor-positive margin rate of 15% to 20%. Consequently, in our study design we included 70 patients, and expected 14 to have a tumor-positive margin. Considering the EGFR overexpression rate of 90% in OSCC, potentially leading to inadequate fluorescence in 10% of the tumors, we expected to detect at least 12 out of 14 tumor-positive margins. Given the sample size, this would result in a sensitivity of 86% (95% CI 60-96%), yielding sufficient precision with regard to the expected impact on real-time intraoperative margin assessment, allowing the informed design of a subsequent comparative randomized study. Estimates of specificity were expected to be even more precise given the predicted larger number of patients with tumor-negative margins.
Data exclusions	Excluded images had the surface of the specimen not perpendicular to the camera, were bone margins, had intraoperative extra resections attached to the specimen prior to fluorescence imaging, or reflection of light in mucosal tissue interfered with evaluation of the resection margin. Exclusion was performed prior to histopathological analysis. Exclusion criteria on based on patient characteristics are described above under "Research involving human participants, their data, or biological material Policy information", in the section "recruitment".
Replication	Image acquisition was not replicated, of all surgical planes one image was obtained. Image analysis was performed by two trained assessors simultaneously for all images. All attempts of image acquisition and analysis were successful.
Randomization	We did not include a control group in the current study, since the primary endpoint was to determine the detection rate of tumor positice margins of our technique.
Blinding	Fluorescence image analysis was performed prior to histology, thus assessors were blinded for outcome of the pathology. Pathologists were blinded for fluorescence imaging analysis during the histopathology process. As described above, no control group was used in the current study, therefore blinding for group allocation was not applicable.

Reporting for specific materials, systems and methods

We require information from authors about some types of materials, experimental systems and methods used in many studies. Here, indicate whether each material, system or method listed is relevant to your study. If you are not sure if a list item applies to your research, read the appropriate section before selecting a response.

Materials & experimental systems

n/a	Involved in the study	
☐	☒	Antibodies
☒	☐	Eukaryotic cell lines
☒	☐	Palaeontology and archaeology
☒	☐	Animals and other organisms
☐	☒	Clinical data
☒	☐	Dual use research of concern
☒	☐	Plants

Methods

n/a	Involved in the study	
☒	☐	ChIP-seq
☒	☐	Flow cytometry
☒	☐	MRI-based neuroimaging

Antibodies

Antibodies used	Patients were administered with cetuximab, and cetuximab-800CW, which is fluorescently labelled cetuximab.
Validation	In the manuscript, we cited the manufacturing progress and reported on earlier research using this antibody in human. Briefly, commercially available cetuximab (Erbitux®) 5 mg/mL was conjugated to IRDye800CW NHS Ester (LI-COR Biosciences, Lincoln, NE, USA) under regulated conditions with a dye:antibody ratio of 2:1. The solution was purified using PD-10 buffer at 1mg/ml exchange columns (GE Healthcare, Chicago, IL, USA). Cetuximab-800CW was formulated in a sodium-phosphate buffer at of 1 mg/mL concentration and sterile filled into injection vials.

Clinical data

Policy information about clinical studies

All manuscripts should comply with the ICMJEguidelines for publication of clinical research and a completedCONSORT checklist must be included with all submissions.

Clinical trial registration	NCT03134846
Study protocol	The full study protocol is included in the submission.
Data collection	Data was collected between Januari 2019 and December 2021. Intraopertive images were obtained in the OR. Pathology data was collected at the pathology lab. Patients and tumor characteristics were taken from the electronic patients file.
Outcomes	The trial's primary endpoints were the FMI detection rate of tumor-positive surgical margins. The secondary endpoints were the detection rates of close surgical margins, the in vivo fluorescence contrast between tumor and adjacent tissue as determined by multi-diameter single-fibre reflectance, single-fibre fluorescence defined as TBRspectroscopy, and the tolerability and safety of the cetuximab-800CW, for which adverse events were graded according to Common Terminology Criteria for Adverse Events (CTCAE) version 5.0.
